# Tunable optical topological transitions of plasmon polaritons in WTe_2_ van der Waals films

**DOI:** 10.1038/s41377-023-01244-w

**Published:** 2023-08-09

**Authors:** Yuangang Xie, Chong Wang, Fucong Fei, Yuqi Li, Qiaoxia Xing, Shenyang Huang, Yuchen Lei, Jiasheng Zhang, Lei Mu, Yaomin Dai, Fengqi Song, Hugen Yan

**Affiliations:** 1 State Key Laboratory of Surface Physics, Key Laboratory of Micro and Nano-Photonic Structures (Ministry of Education), and Department of Physics, Fudan University, 200433 Shanghai, China; 2 Centre for Quantum Physics, Key Laboratory of Advanced Optoelectronic Quantum Architecture and Measurement (MOE), School of Physics, Beijing Institute of Technology, 100081 Beijing, China; 3 Beijing Key Lab of Nanophotonics & Ultrafine Optoelectronic Systems, School of Physics, Beijing Institute of Technology, 100081 Beijing, China; 4 National Laboratory of Solid State Microstructures, Collaborative Innovation Center of Advanced Microstructures, and College of Physics, Nanjing University, 210093 Nanjing, China; 5 Atom Manufacturing Institute (AMI), 211805 Nanjing, China; 6 Center for Superconducting Physics and Materials, National Laboratory of Solid State Microstructures and Department of Physics, Nanjing University, 211805 Nanjing, China

**Keywords:** Nanophotonics and plasmonics, Terahertz optics, Polaritons, Infrared spectroscopy

## Abstract

Naturally existing in-plane hyperbolic polaritons and the associated optical topological transitions, which avoid the nano-structuring to achieve hyperbolicity, can outperform their counterparts in artificial metasurfaces. Such plasmon polaritons are rare, but experimentally revealed recently in WTe_2_ van der Waals thin films. Different from phonon polaritons, hyperbolic plasmon polaritons originate from the interplay of free carrier Drude response and interband transitions, which promise good intrinsic tunability. However, tunable in-plane hyperbolic plasmon polariton and its optical topological transition of the isofrequency contours to the elliptic topology in a natural material have not been realized. Here we demonstrate the tuning of the optical topological transition through Mo doping and temperature. The optical topological transition energy is tuned over a wide range, with frequencies ranging from 429 cm^−1^ (23.3 microns) for pure WTe_2_ to 270 cm^−1^ (37.0 microns) at the 50% Mo-doping level at 10 K. Moreover, the temperature-induced blueshift of the optical topological transition energy is also revealed, enabling active and reversible tuning. Surprisingly, the localized surface plasmon resonance in skew ribbons shows unusual polarization dependence, accurately manifesting its topology, which renders a reliable means to track the topology with far-field techniques. Our results open an avenue for reconfigurable photonic devices capable of plasmon polariton steering, such as canaling, focusing, and routing, and pave the way for low-symmetry plasmonic nanophotonics based on anisotropic natural materials.

## Introduction

Hyperbolic polaritons are a unique type of polariton that exhibits hyperbolic isofrequency contours (IFCs). They are advantageous over traditional isotropic or elliptic polaritons. With extreme anisotropy, the propagation of hyperbolic polaritons is highly directional^1–5^. Meanwhile, they exhibit intense confinement and strong field enhancement, which enable sub-wavelength control of light–matter interactions, making them ideal candidates for applications such as sensing and energy conversion^6^. In addition, the open geometry of the IFC in the momentum space leads to the theoretically infinite wavevectors and unprecedentedly high photonic density of states, which is particularly appealing in quantum applications like the enhancement of spontaneous emission^7^.

Hyperbolic polaritons are typically found in man-made metamaterials, which require complicated nanofabrication^8,9^. Fortunately, some anisotropic materials in nature have been discovered to host in-plane hyperbolic polaritons, such as phonon polaritons in MoO_3_^10,11^ and V_2_O_5_^12^, and plasmon polaritons in WTe_2_^13^, which open up a plethora of opportunities in reconfigurable on-chip integrated photonics^14,15^. These findings have fueled an emerging research field termed low-symmetry nanophotonics^16–19^. Crucially, the tunability of the wavelength range of the hyperbolic IFCs (the hyperbolic regime) and the optical topological transition (OTT) of the IFCs to the elliptic topology^20^ are highly desirable to fulfill the potential. However, phonon polaritons, which are based on polar lattice vibrations, intrinsically show rather fixed Reststrahlen bands and material-specific polariton dispersions. This certainly limits their tunability, although much efforts have been devoted to extrinsic schemes in MoO_3_ phonon polaritons, such as interfacing with suitable substrate^21–24^, stacking with highly tunable graphene^25–29^, and twisting bilayer structures^30–33^. Progress has also been achieved through more disruptive measures, such as intercalation^12,34^, but the phonon bands only shift a small amount^12^ (around 30 cm^−1^), and hyperbolic polariton was not observed after intercalation.

On the other hand, plasmon polaritons, particularly in the two-dimensional form, are easier to be tamed intrinsically^35,36^, which has been exemplified by graphene plasmon polaritons^37^. However, naturally existing in-plane hyperbolic plasmon polaritons are rare, but have recently been demonstrated in WTe_2_ thin films^13^ in the frequency range of 429–632 cm^−1^, with the elliptic regime of the IFCs below 429 cm^−1^. Given the limited options for such materials, it’s even more imperative to tune the OTT energy to suit various applications. In fact, the plasmon dispersion in a natural hyperbolic plasmonic surface is typically governed by the anisotropy of both free carrier response and bound interband transitions^13,38^, with the former for inductive and the latter for capacitive optical responses. Any variation of electronic properties, such as carrier density, effective mass anisotropy, frequency and strength of interband transition resonance, will give rise to a modulation of the hyperbolic regime^38,39^. Though as promising as it sounds, however, the experimental demonstration of tunable in-plane OTT of IFCs of plasmon polaritons in a natural material has not been realized up to date.

In this study, we report the first intrinsic tuning of such OTT in a broad wavelength range through chemical doping and temperature in a natural material. We reveal such tunability in Mo-doped WTe_2_ (Mo_*x*_W_1-*x*_Te_2_) thin films, a recently discovered layered Type-II Weyl semimetal with composition-dependent band structure^40,41^ and electric transport^42^. Meanwhile, an innovative technique to track the topology based on the far-field polarization dependence of the localized surface plasmon resonances (LSPRs) in skew ribbons has been developed. This technique allows for efficient and accurate characterization of the topology of plasmon dispersion (IFCs) at a particular frequency in a single sample. Our study not only extends the hyperbolic regime in natural materials by other degrees of freedom, but also reveals the peculiar and informative polarization property of LSPRs in microstructures made from an anisotropic material.

## Results

### Sample fabrication and polarized IR spectra

**Fig. 1:**
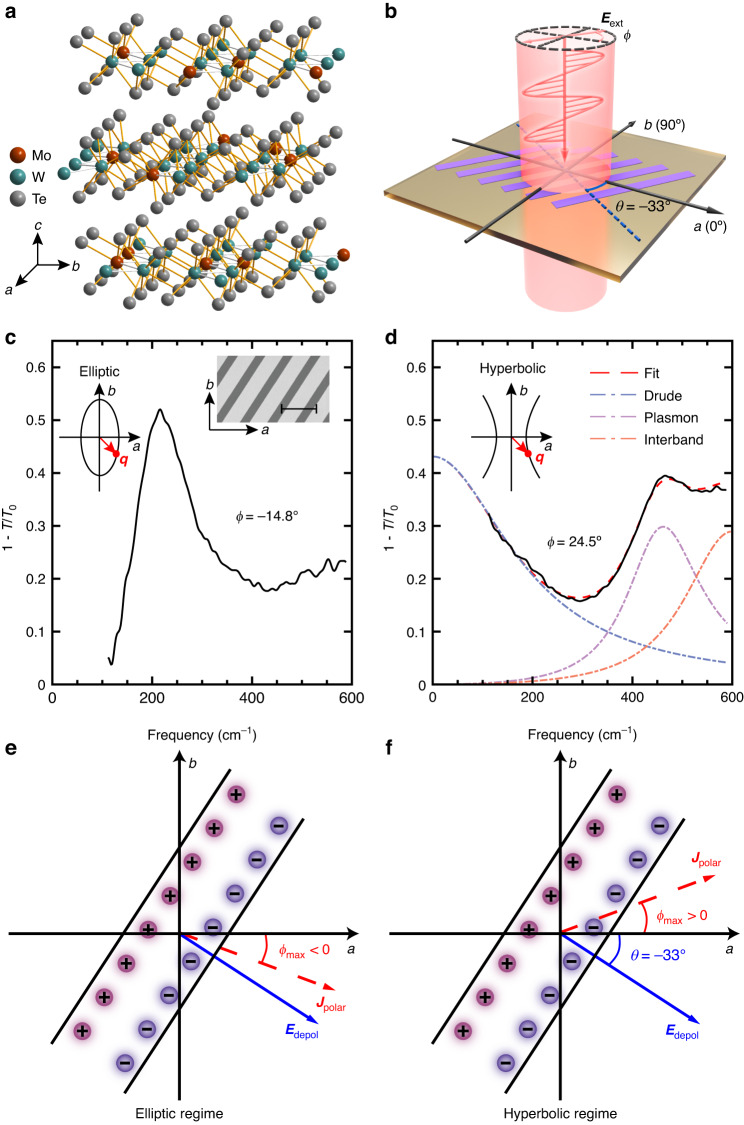
Polarization *ϕ*_max_ for the maximal plasmon intensity. **a** A sketch of Mo-doped WTe_2_ in *T*_*d*_-phase. Brown/green represents Mo/W atoms, gray represents Te atoms. **b** Schematic illustration of skew ribbon arrays under illumination. Counterclockwise rotation with respect to *a*-axis is defined as positive. The polarization of normal incident light is *ϕ* and the perpendicular direction of the ribbon is $\theta=-33^\circ$. **c**, **d** The typical extinction spectra of skew ribbon arrays (composition ratio *x* = 0.278, ribbon widths of 4.5 and 1.9 μm) along the polarization ($\phi=-14.8^\circ, \,24.5^\circ$) in the elliptic/hyperbolic regime, respectively. Right inset in **c** shows the SEM image of the skew ribbon array. Scale bar is 10 μm. Top left insets in (**c**, **d**) are schematics of the IFCs of plasmon mode in the elliptic and hyperbolic regimes respectively. Red arrows labeled by ***q*** are the corresponding wavevector direction (related to the −33° skew angle). **e**, **f** The configuration of polarization current density ${{\boldsymbol{J}}}_{{\rm{polar}}}$ and depolarization field ${{\boldsymbol{E}}}_{{\rm{depol}}}$ in elliptic and hyperbolic regimes, respectively. ${\phi }_{{\rm{\max }}}$ has the same sign as *θ* in the elliptic regime, but the opposite sign in the hyperbolic regime

We grew Mo_*x*_W_1-*x*_Te_2_ crystals (*x* ≤ 0.5) in the semi-metallic orthorhombic *T*_*d*_-phase using a chemical vapor transport technique with iodine as the transport agent (“Materials and methods” and Supplementary Note 1). The zigzag W-W chains are along *a*-axis, with W atoms partially substituted by Mo after Mo doping^43^, as displayed in Fig. 1a. Figure 1b shows the schematic illustration of a skew ribbon array patterned from an exfoliated single crystal film of Mo_*x*_W_1-*x*_Te_2_ with a skew angle of $\theta=-33^\circ$ with respect to *a-*axis (“Materials and methods”), and illuminated by the normal incident light with a polarization angle *ϕ*. LSPRs can be excited in such ribbon arrays with far-field incident light. Figure 1c, d displays two typical extinction spectra (characterized by $1-T/{T}_{0}$, where *T* and ${T}_{0}$ are the transmission of light through the sample and the bare substrate, respectively) of such skew ribbon arrays with composition ratio *x* = 0.278 but different ribbon widths *L* (corresponding to the effective plasmon wavevector of ${\rm{\pi }}/L$). The light polarization (*ϕ* = −14.8°, 24.5°) was selected as close as possible to where the plasmon resonance is most intense, with resonance frequencies (219 cm^−1^, 462 cm^−1^) in the elliptic and the hyperbolic regimes respectively (to be discussed below). Besides the plasmon resonance, the spectrum in Fig. 1d exhibits evident Drude response, in sharp contrast to that in Fig. 1c, suggesting that the polarization for maximal plasmon intensity in the hyperbolic regime deviates significantly from the perpendicular direction of ribbons. Such deviation was first reported in self-assembled carbon nanotubes^44^. However, the implication on the topology of plasmon dispersion has not been revealed. Here, we show that such optimal polarization is fully dictated by the ratio of the imaginary parts of the anisotropic conductivities, and in turn can be utilized to determine the topology of plasmon dispersion.

### Polarization dependence of LSPRs in skew ribbon arrays

When a skew ribbon array is illuminated, the plasmon resonance is most intense when the polarization of the incident light ${{\boldsymbol{E}}}_{{\rm{ext}}}$ is parallel to the polarization current density ${{\boldsymbol{J}}}_{{\rm{polar}}}$ (${{\boldsymbol{J}}}_{{\rm{polar}}}=\partial {\boldsymbol{P}}/\partial t$, with ${\boldsymbol{P}}$ as the polarization vector), which is associated with the depolarization field ${{\boldsymbol{E}}}_{{\rm{depol}}}$ induced by the polarization charge. Note that ${{\boldsymbol{E}}}_{{\rm{depol}}}$ is always perpendicular to the ribbon edge due to the translation invariance of the polarization charge distribution along the edge (Fig. 1e, f), and ${{\boldsymbol{J}}}_{{\rm{polar}}}$ is not the conduction current related to the real part of the conductivity, which is responsible for the energy dissipation in the material. In an isotropic two-dimensional material, the incident light with polarization perpendicular to the ribbon leads to the maximal plasmon resonance, since ${\boldsymbol{P}}$ and hence ${{\boldsymbol{J}}}_{{\rm{polar}}}$ are parallel to ${{\boldsymbol{E}}}_{{\rm{depol}}}$ due to the isotropic polarizability (conductivity) tensor. In ribbons made from an anisotropic film, however, ${\boldsymbol{P}}$ and ${{\boldsymbol{J}}}_{{\rm{polar}}}$ are not necessarily parallel to ${{\boldsymbol{E}}}_{{\rm{depol}}}$. Thus, as shown in Fig. 1e, f, the optimal polarization ${\phi }_{{\rm{\max }}}$ for plasmon excitation deviates from the perpendicular direction of the ribbon, the value of which is determined by the ratio of the imaginary parts of conductivities (${\sigma }_{{aa}}^{{\rm{\text{'}\text{'}}}}$ and ${\sigma }_{{bb}}^{{\rm{\text{'}\text{'}}}}$) and the skew angle $\theta$ (Supplementary Note 2):

1 $${\rm{tan }}\,{\phi }_{{\rm{max }}}\left(\omega \right)=\frac{{\sigma }_{{bb}}^{\prime\prime}\left(\omega \right)}{{\sigma }_{{aa}}^{\prime\prime}\left(\omega \right)}{\rm{tan }}\,\theta$$

where ${\phi }_{\max }$ can be restricted in the range of −90° to 90°. Therefore, ${\phi }_{\max }$ has the same sign as *θ* in the elliptic regime since ${\sigma }_{{aa}}^{\prime\prime}{\sigma }_{{bb}}^{\prime\prime}\, >\, 0$ (Fig. 1e), but the opposite sign in the hyperbolic regime, for which ${\sigma }_{{aa}}^{\prime\prime}{\sigma }_{{bb}}^{\prime\prime}\, <\, 0$ (Fig. 1f). Particularly, for the plasmon polaritons at the OTT energy (${\sigma }_{{bb}}^{{\prime\prime}}=0$), the optimal light polarization coincides with *a*-axis.

**Fig. 2:**
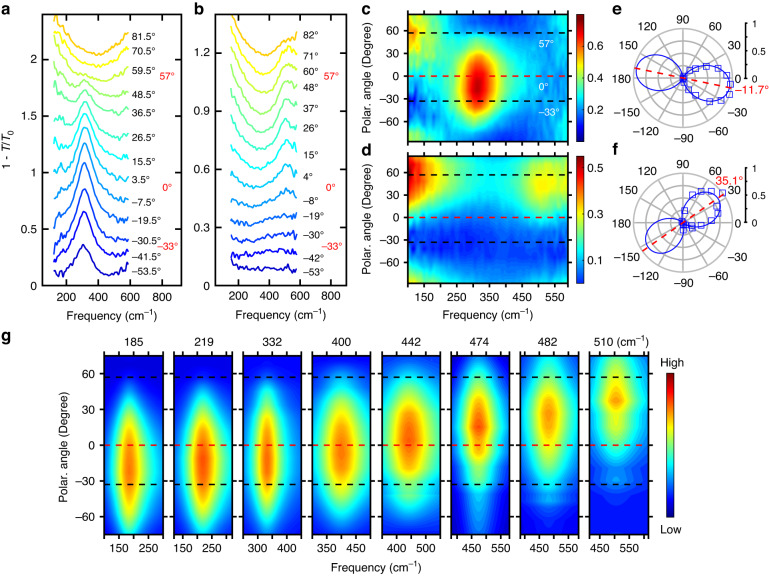
Polarization-resolved LSPRs in skew ribbon arrays of WTe_2_. **a**, **b** Polarization dependence of raw extinction spectra at plasmon frequencies of 308 cm^−1^ and 510 cm^−1^, respectively. Degrees in black indicate the polarization of incident light. Angles of 57° and −33° in red represent the parallel and perpendicular directions of ribbon edges. 0° denotes *a*-axis of WTe_2_. For clarity, spectra are shifted vertically. **c**, **d** The corresponding pseudocolor maps of (**a**, **b**). **e**, **f** Polarization dependence of the corresponding normalized plasmon weights of (**a**, **b**). Blue solid lines are fitting results of cos^2^ $\phi$. **g** The pseudocolor maps of the plasmon absorption spectra at different resonance frequencies. **c**, **d**, **g** black dashed lines denote the parallel (57°) and perpendicular (−33°) directions with respect to the ribbon and the red dashed lines denote *a*-axis

To benchmark this scheme, we firstly use Eq. (1) to reexamine the topology of plasmon dispersion in WTe_2_ films, for which an OTT has been reported at about 429 cm^−1^ (23.3 microns in wavelength)^13^. Skew ribbon arrays as in Fig. 1b with the same skew angle of *θ* = −33° but different ribbon widths were fabricated from WTe_2_ films. It should be noted that all of the studied skew ribbons in this paper have the same −33° skew angle (Supplementary Note 3). The polarization-resolved extinction spectra for two representative samples at 10 K are shown in Fig. 2a, b (“Materials and methods”). Resonance peaks of LSPRs can be observed at frequencies of 308 cm^−1^ in the elliptic regime (Fig. 2a) and 510 cm^−1^ in the hyperbolic regime (Fig. 2b), thus expected to have maximal plasmon intensity at polarization angles of different signs, according to Eq. (1). Note that, in Fig. 2b, spectra with polarization at around −33° (perpendicular to the ribbon) are nearly flat, indicating almost no plasmon absorption, in striking contrast to ribbons patterned from isotropic films. To see the polarization dependence more clearly, the extinction spectra in Fig. 2a, b are plotted as pseudocolor maps in Fig. 2c, d. The maximal plasmon intensity in Fig. 2c can be found below the zero line (red dashed line), while the plasmon resonance in Fig. 2d exhibits strongest absorption at an angle well above zero. The feature below 200 cm^−1^ comes from the Drude response of free carriers, whose maximum is always along the ribbon (*ϕ* = 57°), a common scenario for both isotropic and anisotropic ribbons (Supplementary Note 4). To extract the plasmon weight, spectra were fitted with the Drude–Lorentz model (“Materials and methods” and Supplementary Note 5), and the fitted plasmon weight is plotted in Fig. 2e, f as a function of the polarization angle. As shown in Fig. 2e (Fig. 2f), the polar angle ${\phi }_{{\rm{\max }}}$ is −11.7° (35.1°), which has the same (opposite) sign as the skew angle (−33°), consistent with the elliptic (hyperbolic) topology of IFCs.

**Fig. 3:**
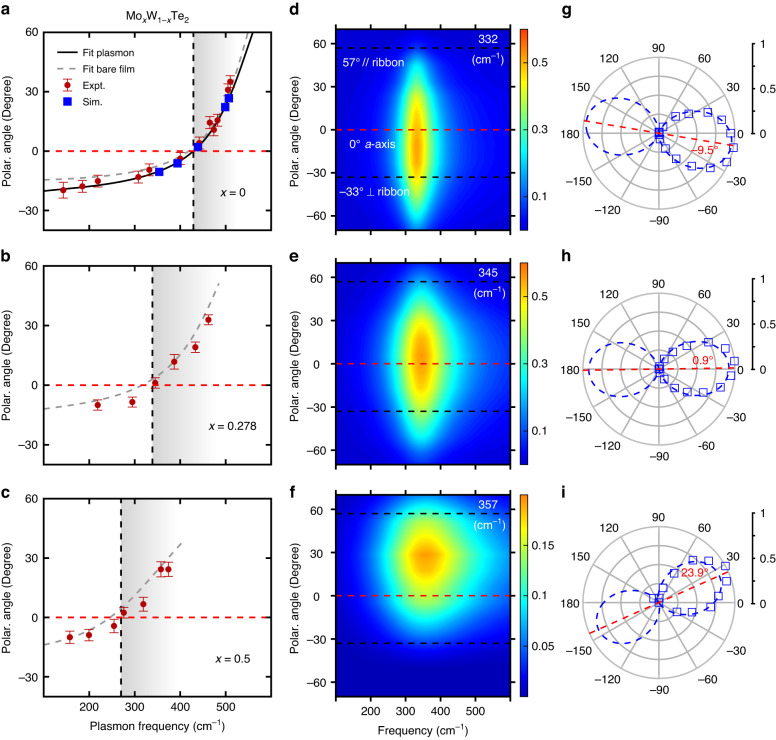
Tunable OTTs by Mo doping. **a**–**c** The polarization ${\phi }_{{\rm{\max }}}$ as a function of the plasmon frequency at different doping levels. Brown dots are fitted results of polarization experiments with errors of about 3° from both angle measurements and fittings. Blue squares are fitted results of simulations of the same configurations (Supplementary Note 6). Vertical black dashed line highlights the lower boundary of the hyperbolic regime. Black solid curve in (**a**) is based on the conductivity from the plasmon dispersion in ref. ^13^. Gray dashed lines in (**a**–**c**) are calculated using the fitted conductivities from the extinction spectra of unpatterned films in Fig. 4a–c. **d**–**f** The pseudocolor maps of the plasmon spectra with similar resonance frequencies (345 ± $15$ cm^−1^) at different Mo-doping levels (0, 0.278, 0.5, from top to bottom). **g**–**i** The corresponding normalized plasmon weights of (**d**–**f**) versus the polarization

More systematically, a series of samples with the same skew angle ($\theta=-33^\circ$) and incremental plasmon frequencies ranging from 185 cm^−1^ to 510 cm^−1^ were fabricated by varying the ribbon width. The polarization-resolved absorption spectra due to the plasmon resonance are plotted as pseudocolor maps for several plasmon frequencies in Fig. 2g (“Materials and methods”). The angle for maximal plasmon intensity gradually evolves from negative to positive, crossing the zero line (*a*-axis) when the plasmon frequency coincides with the elliptic/hyperbolic boundary (429 cm^−1^, determined by the plasmon dispersion in previous work^13^). The measured frequency-dependent polarization angle for the maximal plasmon intensity (*ϕ*_max_) is displayed in Fig. 3a as brown dots, with errors from both angle measurements and fittings (Supplementary Note 5). In addition, the simulations of the extinction spectra in WTe_2_ skew ribbon arrays ($\theta=-33^\circ$) with different ribbon widths were performed (Supplementary Note 6). The fitted results of the optimal ${\phi }_{{\rm{\max }}}$ are displayed as blue squares in Fig. 3a. The OTT of IFCs from the elliptic to the hyperbolic can be directly manifested by the sign change of the angle based on experiments and simulations, which agrees well with the calculations by Eq. (1) (black solid line) using the conductivities extracted from the plasmon dispersion in previous work^13^ (Supplementary Note 7), validating the capability of tracking the OTT both qualitatively and quantitatively. Note that when the skew angle is fixed, ${\phi }_{{\rm{\max }}}$ is solely determined by $\frac{{\sigma }_{{bb}}^{{\rm{\text{'}\text{'}}}}\left(\omega \right)}{{\sigma }_{{aa}}^{{\rm{\text{'}\text{'}}}}\left(\omega \right)}$ at the plasmon frequency and will not be affected by the ribbon width or film thickness (assuming an unchanged band structure with sample thickness), enabling it to be a superior method to determine the topology of plasmon dispersion at individual frequencies.

### Mo-doping-dependent OTT of IFCs of plasmon polaritons

With the convenient toolkit at our disposal, we can now proceed to investigate the tuning of such OTT. Ribbon arrays with the same configuration as shown in Fig. 1b were fabricated on Mo-doped WTe_2_ films with various ribbon widths, resulting in different plasmon frequencies (Supplementary Note 8). The polarization dependence of plasmon polaritons was measured and the fitted ${\phi }_{{\rm{\max }}}$ values are summarized in Fig. 3b–c for 27.8% and 50% doping levels, respectively. A sign change for ${\phi }_{{\rm{\max }}}$ can be observed in Fig. 3b–c as the plasmon frequency increases, indicating an OTT in the doped samples as well. A large redshift of the zero-crossing point or the OTT frequency (indicated by the vertical black dashed line) occurs. It changes from 429 cm^−1^ (23.3 microns) for WTe_2_ to about 270 cm^−1^ (37.0 microns) for Mo_0.5_W_0.5_Te_2_. This corresponds to a 38% redshift in frequency or a 1.6-fold increase in wavelength for the OTT energy.

**Fig. 4:**
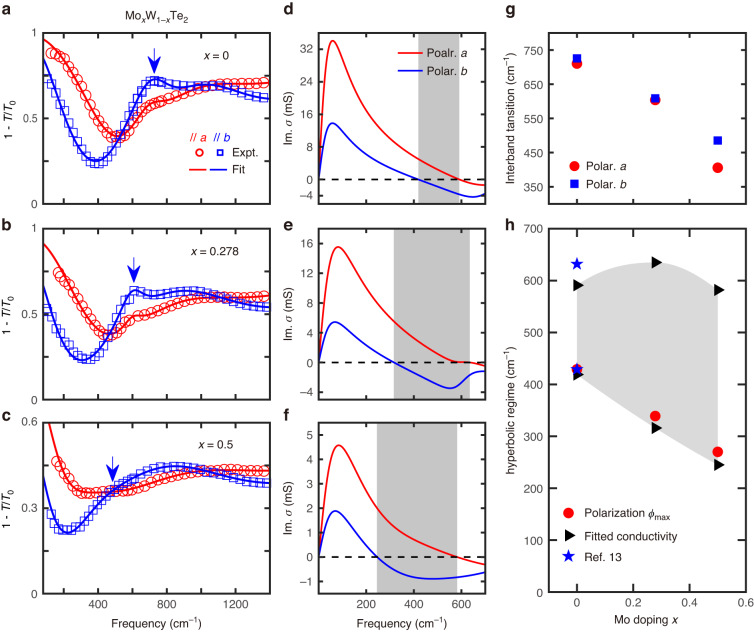
The mechanism of tunable OTTs by Mo doping. **a**–**c** The experimental and fitting results of anisotropic extinction spectra of bare films of Mo_*x*_W_1-*x*_Te_2_. Blue downward arrows represent the first interband transition resonances along *b*-axis. **d**–**f** The imaginary parts of conductivities along two crystal axes. Shaded areas denote the corresponding hyperbolic regimes. **g** Doping dependence of the frequency of the first interband transition resonance. **h** Doping dependence of the hyperbolic regime. Black right-pointing triangles are determined by fitted conductivities from (**d**–**f**) and shaded areas are the corresponding hyperbolic regimes. Red dots are determined by the polarization ${\phi }_{{{\max }}}=0^\circ$. Blue pentagrams are from the plasmon dispersion in ref. ^13^

To further manifest the doping effect on the OTT, as an example, we compare the polarization dependence of plasmon polaritons with similar resonance frequencies of 345 ± 15 cm^−1^ for different doping levels. As shown in Fig. 3d–f, the polarization angle for the maximal plasmon intensity increases from negative to positive upon doping. This can be seen even more clearly in Fig. 3g–i, which shows that the angle ${\phi }_{{\rm{\max }}}$ increases from –9.5° for WTe_2_ films to 23.9° at 50% Mo doping, suggesting the OTT of IFCs from the elliptic to the hyperbolic by Mo doping. Note that the plasmon linewidth of these samples increases with doping (72 cm^−1^ of WTe_2_, 112 cm^−1^ at 27.8% doping and 250 cm^−1^ at 50% doping). The increasing linewidth is primarily due to the stronger coupling between plasmon polaritons and interband transitions, which have lower energy at larger doping levels and hence are closer to the plasmon mode, as shown in Fig. 4g. Such coupling-induced broadening is manifested in WTe_2_ as well^13^. As shown in Fig. 2g, the plasmon linewidth increases as the frequency approaches that of the interband transition. The linewidth of intrinsic plasmon resonances at lower frequencies, hence largely free of interband coupling, is more similar for all Mo_*x*_W_1-*x*_Te_2_ samples (with resonance frequencies of 295.0, 291.7, 214.3 cm^−1^ and quality factors of 3.4, 3.1, 2.1 along *a*-axis at different doping levels respectively, Supplementary Note 9).

### Hyperbolic regime determined by the optical absorption in Mo_*x*_W_1-*x*_Te_2_ thin films

To delve into the physical mechanism of the tunable OTT, polarization-resolved extinction spectra of pristine films of Mo_*x*_W_1-*x*_Te_2_ (*x* = 0, 0.278, 0.5, with thicknesses of 110, 80, 42 nm, respectively) were examined. The measured spectra (far- and mid-IR ranges) and their fitting curves (Supplementary Note 10) are plotted in Fig. 4a–c. The corresponding extracted sheet optical conductivity (imaginary part) is displayed in Fig. 4d–f, with the generally expanding hyperbolic regime marked by the shaded area based on their signs. The IFCs of plasmon dispersion in Mo_*x*_W_1-*x*_Te_2_ are also plotted according to the loss function^13^ to visualize the OTT (Supplementary Note 11). The lower boundary of the hyperbolic regime redshifts upon doping, consistent with the polarization behavior of LSPRs in skew ribbon arrays. In general, the topology of plasmon dispersion is determined by the optical response of both the intraband (Drude response) and interband transitions of carriers. The transition energy and oscillator strength of the interband transition resonance are determined by the specific band structure and the Fermi level, and the free carrier Drude response depends on the carrier density and effective mass. In our experiment, the extinction spectra in Fig. 4a–c share similar qualitative profiles. The primary difference in the far-IR spectra is quantitative in nature, such as the peak position of the first interband transition resonance, as shown in Fig. 4g. The transition energy decreases from 726 cm^−1^ for WTe_2_ to about 488 cm^−1^ at 50% doping (Supplementary Note 10), as indicated by blue arrows for the spectra of *b*-axis polarization in Fig. 4a–c, which brings the whole interband transition feature to lower frequencies. This salient doping dependence governs the redshift of the hyperbolic lower boundary, as the dielectric (capacitive) part of the optical conductivity at long wavelength is primarily attributed to interband transitions. It is also worth noting that the fitted Drude weight (normalized with thickness) along *b*-axis slightly decreases upon Mo doping (Supplementary Note 10), playing a minor role in the redshift of the OTT energy.

Furthermore, by substituting Im(*σ*) in Fig. 4d–f into Eq. (1), the calculated frequency dependence of ${\phi }_{{\rm{\max }}}$ is plotted as gray dashed lines in Fig. 3a–c, which are consistent with the directly measured angles (brown dots) in skew ribbon arrays. Hyperbolic boundary frequencies derived from the polarization dependence of skew ribbons (red dots), film extinction measurements (black right-pointing triangles), and the plasmon dispersion in ref. ^13^ (blue pentagrams) are summarized in Fig. 4h. All these procedures show consistent doping dependence of the lower boundary, substantiating the redshift of the OTT energy.

### Temperature-dependent OTT of IFCs of plasmon polaritons

**Fig. 5:**
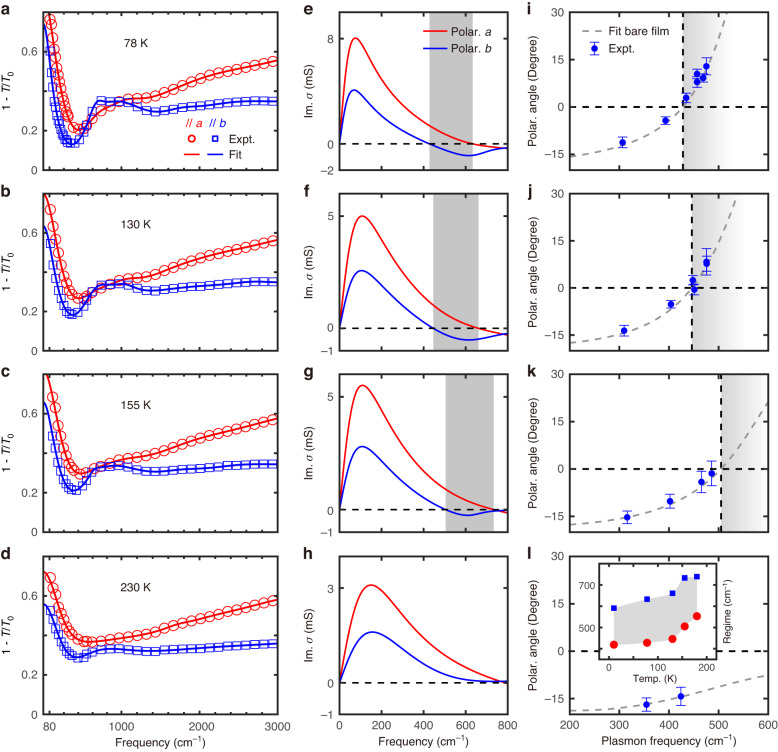
Temperature-induced shifts of OTT energy in WTe_2_. **a**–**d** The anisotropic extinction spectra and fitting curves of a bare film of WTe_2_ at 78, 130, 155, and 230 K, respectively. **e**–**h** The corresponding extracted imaginary parts of the optical conductivities along two crystal axes in (**a**–**d**). The shaded area represents the hyperbolic regime. **i**–**l** The optimal polarization ${\phi }_{{\rm{\max }}}$ as a function of the plasmon frequency at 78, 130, 155, and 230 K, respectively. Dashed lines are calculated using the fitted optical conductivities in (**e**–**h**) at the corresponding temperature. Inset in (**l**) shows the temperature dependence of the hyperbolic regime determined by the corresponding extracted imaginary part of the optical conductivity (Supplementary Note 12)

As a typical semimetal, WTe_2_ exhibits strong temperature dependence in its electronic properties^13,45^, enabling active tuning of the OTT. To fulfill this potential, the polarization-resolved extinction spectra of a 35-nm-thick WTe_2_ bare film at different temperatures (78 K, 130 K, 155 K, 180 K, 230 K, 300 K) were measured. The corresponding spectra (far- and mid-IR ranges) and fitting curves are plotted in Fig. 5a–d. The anisotropy of interband transitions (between 700 and 1100 cm^−1^) along two axes decreases, whereas the Drude weights increase due to more thermal carriers at higher temperatures. As a result, the lower boundary of the hyperbolic regime blueshifts from 428 cm^−1^ at 78 K to 553 cm^−1^ at 180 K, which is manifested by the extracted sheet optical conductivity in Fig. 5e–h and the temperature dependence of IFCs (Supplementary Note 11). The hyperbolic regime in the far-IR range vanishes above 230 K. A kink appears in the temperature dependence of the hyperbolic regime at about 130 K (inset in Fig. 5l), which is consistent with the temperature dependence for the plasmon frequency and Drude weight in WTe_2_^13^. This is likely attributed to the temperature-induced Lifshitz transition at about 147–160 K, where the two hole pockets move down in energy with respect to the Fermi surface and eventually disappear, resulting in no hole carriers at higher temperatures^46,47^. Further, the polarization dependence of plasmon polaritons of the same devices as those shown in Fig. 3a at corresponding temperatures was measured. The polarization angle ${\phi }_{{\rm{\max }}}$ was extracted in the same way, which agrees well with the calculation of Eq. (1) as shown in Fig. 5i–l, demonstrating the temperature-induced shifts of the OTT energy. Additional details are summarized in Supplementary Note 12. Compared to nearly temperature-independent phonon polaritons, the hyperbolic regime in WTe_2_ plasmon polaritons shifts over a wide range with temperature. By combining Mo doing and temperature tuning, the hyperbolic regime overall covers the far-IR spectrum from 13.5 microns (739 cm^−1^) to 37.0 microns (270 cm^−1^), which is 3.1 times broader than the hyperbolic wavelength range observed in pristine WTe_2_ films at 10 K^13^.

## Discussion

### Characterization of the OTT in the far-IR range with the far-field method

As a matter of fact, determining the topology of plasmon dispersion in the far-IR range is a daunting task without the aforementioned polarization-based method. In principle, both near- and far-field techniques can probe the OTT in WTe_2_. However, the near-field scheme is not mature in the far-IR range, especially with samples in the cryogenic conditions, even though it is widely and successfully employed in the mid-IR range to image in-plane hyperbolic phonon polaritons^3–5,10–12,16,21–23,25–34^. As a consequence, up to now, there is no near-field imaging of hyperbolic plasmon polaritons in WTe_2_. As for the far-field technique, previously we determined the OTT of plasmon polaritons in WTe_2_ through mapping the plasmon dispersion in the whole two-dimensional momentum space^13^, which was laborious and required numerous samples (each momentum ***q*** needs a ribbon array). Fortunately, with our polarization-based far-field method, we can now determine the topology of the IFC at a particular frequency in a single sample without invoking the whole plasmon dispersion, which is truly advantageous.

### Implications of the tuning of the OTT in WTe_2_

By leveraging Mo doping and temperature, the hyperbolic regime expands 3.1 times than that in pristine WTe_2_. This significant broadening demonstrates that hyperbolic plasmon polaritons can be manipulated more readily, which is fundamentally different from previously reported hyperbolic phonon polaritons. For instance, the fabrication of MoO_3_/MoO_3_ twisted bilayers leads to a contraction of the hyperbolic regime^30–33,48^. As a result, the expanded hyperbolic regime now covers nearly the entire far-IR range. This expansion complements the existing hyperbolic phonon polaritons that predominantly reside in the mid-IR range. Moreover, the far-IR range contains a multitude of intramolecular or intermolecular vibration modes (e.g., in proteins or DNA), making Mo_*x*_W_1-*x*_Te_2_ an excellent candidate for bio-sensing and bio-imaging applications^49^. Furthermore, both the lower and higher boundaries of the hyperbolic regime, which correspond to the sigma-near-zero points along *b*- and *a*-axis, respectively, experience substantial shifts. When a material exhibits near-zero effective permittivity (conductivity), novel physical effects arise, such as field enhancement, tunneling through anomalous waveguides and transmission with small phase variation, which are also known as epsilon-near-zero photonics^50^. Thus, Mo_*x*_W_1-*x*_Te_2_ naturally serves as an in-plane tunable anisotropic sigma-near-zero material for functional photonic devices.

Particularly, at the lower hyperbolic boundary, the IFC comprises two nearly parallel lines along *b-*axis in the two-dimensional momentum space, analogous to the so-called canalization regime in hyperbolic phonon polaritons^3,30,32^. Thus, the tunability of the lower boundary allows for the canalization of the energy flow of hyperbolic polaritons over a wide spectral range. The estimated propagation length of canalized plasmon polaritons in WTe_2_ reaches 0.5 micron^13^, which is comparable to that of isotropic plasmon polaritons in graphene^51^ and that of the canalized phonon polaritons in h-BN metasurfaces^3^. In addition, the doping procedure does not degrade the sample quality much, as suggested by the similar figure of merits (Supplementary Note 9). The lifetime of plasmon polaritons in Mo_*x*_W_1-*x*_Te_2_ is ~0.05 picosecond (ps), comparable to that of plasmon polaritons in the undoped WTe_2_ (0.1 ps)^13^ and in graphene on SiO_2_/Si substrates (about 0.05–0.1 ps)^52^, though it is smaller than the lifetime of phonon polaritons in MoO_3_ (about 8 ps)^10^. Future efforts can be devoted to increasing the sample quality through more meticulous growth and judicious choice of substrates.

In conclusion, our work demonstrates the inherent tunability of hyperbolic plasmon polaritons and the OTT in vdW surfaces by chemical doping and temperature over a wide range. The tuning mechanism involves both bound states and free carriers, providing more dimensions for manipulating OTT. Our experiments leverage a unique feature in the polarization-resolved extinction spectra of skew ribbons to determine the topology of IFCs, which can be of great use to investigate other anisotropic two-dimensional materials.

## Materials and methods

### Mo_*x*_W_1-*x*_Te_2_ crystal growth

Mo_*x*_W_1-*x*_Te_2_ single crystals were grown by a chemical vapor transport technique with iodine as the transport agent. Stoichiometric mixtures of Mo, W, and Te powders were loaded into a quartz tube along with a small amount of iodine, which was subsequently sealed in vacuum and placed in a two-zone furnace. The hot zone was maintained at 850 °C for 2 weeks, while the cold zone was kept at 750 °C. The composition of the final crystal was characterized using energy dispersive spectroscopy (EDS) with a scanning electron microscope.

### Sample preparation and fabrication

Single crystal WTe_2_ was bought from HQ Graphene. Bare films of Mo_*x*_W_1-*x*_Te_2_ (*x* ≤ 0.5) with thickness about 40–120 nm through the standard exfoliation technique were transferred to polycrystalline diamond substrates. The substrate has no polar phonon absorption and the transmission is about 70% in the far-IR range. The thickness of the sample was determined by a stylus profiler (Bruker DektakXT) in conjunction with the optical contrast. The preferred sample size is 200 by 200 μm^2^, with the side length longer than the far-IR wavelength (~100 μm). Skew ribbon arrays were patterned using electron beam lithography (Zeiss Sigma SEM with Raith Elphy Plus), with the uncertainty in the skew angle of less than 0.5°. An intermediate skew angle of −33° was chosen in our study, because a too-small angle results in a small ${\phi }_{{\rm{\max }}}$ as shown in Eq. (1), and a too large one leads to a more dramatic reduction of the maximal plasmon frequency achievable in that wavevector direction. Reactive ion etching (RIE) with SF_6_ gas was used to define the ribbons. If necessary, the surroundings of the sample were further etched away using the director writer UPG501 and RIE to ensure IR response only from the targeted sample.

### Far-IR optical spectroscopy

For the polarized far-IR extinction spectra, we used a Bruker FTIR spectrometer (Vertex 70 v) integrated with a Hyperion 2000 microscope and a cryogen-free silicon bolometer system as the detector. The incident light was focused on Mo_*x*_W_1-*x*_Te_2_ samples with a 15× IR objective. A THz polarizer was used to control the light polarization. The samples were cooled to 10 K in a helium-flow cryostat (Janis Research ST-300) with vacuum at about $5$ × 10^−5^ $\text{mbar}$. Throughout the entire measurements, compressed dry air with dew point below −70 °C was purged to an enclosed space housing the cryostat. This procedure minimized the absorption of IR light by moisture in air and effectively increased the signal/noise ratio. The polarization dependence of plasmon polaritons in skew ribbon arrays was studied by rotating the polarizer with a step size of 11.3° from −90° to 90°. Hence, a total of 16 spectra were collected to extract each ${\phi }_{{\rm{\max }}}$.

### Fitting of IR extinction spectra and plotting pseudocolor maps for plasmon spectra

The extinction spectrum is determined by the sheet optical conductivity $\sigma \left(\omega \right)$ as follows^52^:

2 $$1-\frac{T}{{T}_{0}}=1-\frac{1}{{\left|1+{Z}_{0}\sigma \left(\omega \right)/\left(1+{n}_{{\rm{s}}}\right)\right|}^{2}}$$

where ${Z}_{0}$ is the vacuum impedance, *ω* is the frequency of light, and ${n}_{{\rm{s}}}$ is the refractive index of the substrate. The conductivity of the sample is expressed by the Drude–Lorentz model, where the Drude model describes free carriers and the Lorentz model accounts for the bound states such as plasmon resonance or interband transitions in our system:

3 $$\sigma \left(\omega \right)=\frac{{\rm{i}}}{{\rm{\pi }}}\frac{D}{\omega+{\rm{i}}\varGamma }+\sum _{{\rm{k}}}\frac{{\rm{i}}}{{\rm{\pi }}}\frac{\omega {S}_{{\rm{k}}}}{{\omega }^{2}-{{\omega }_{{\rm{k}}}}^{2}+{\rm{i}}{\omega \varGamma }_{{\rm{k}}}}$$

In Eq. (3), *D* and ${S}_{{\rm{k}}}$ represent the spectrum weights, ${\omega }_{{\rm{k}}}$ represents the resonance frequency of the plasmon resonance or interband transitions, *Γ* and ${\varGamma }_{{\rm{k}}}$ are the corresponding FWHMs. The polarization angle ${\phi }_{{\rm{\max }}}$ was extracted by fitting the whole set of 16 polarization spectra with one Drude component and two Lorentz components (plasmon resonance, interband transitions, respectively). The spectral weights were fitting parameters and the Drude scattering rate, plasmon frequency and interband transition resonance frequency were kept the same for all spectra. Pseudocolor maps in Fig. 2g and Fig. 3d–f were plotted by substituting the fitted conductivities of plasmon resonances into Eq. (2) in turn. The plasmon weights ${S}_{{\rm{p}}}$ extracted above were fitted as cos^2^ $\phi$ to obtain ${\phi}_{{{\max}}}$. The fitting details of the extinction spectra of Mo_*x*_W_1-*x*_Te_2_ bare films in Fig. 4a–c are discussed in Supplementary Note 10.

### Supplementary information

Supplementary Information

## Data Availability

All data needed to evaluate the conclusions in the paper are present in the main text and the supplementary information.
