# Supplementary material for: Tunable optical topological transitions of plasmon polaritons in WTe2 van der Waals films
Source: Light Sci Appl. 2023 Aug 9;12:193. doi: 10.1038/s41377-023-01244-w (PMC10409815; doi:10.1038/s41377-023-01244-w)
Supplement: Supplementary file 1 — Supplementary Information [file 41377_2023_1244_MOESM1_ESM.docx]

**Supplementary Information for**

**Tunable optical topological transitions of plasmon polaritons in WTe_2_ van der Waals films**

Yuangang Xie^1^, Chong Wang^2,3^*, Fucong Fei^4,5^*, Yuqi Li^2,3^, Qiaoxia Xing^1^, Shenyang Huang^1^, Yuchen Lei^1^, Jiasheng Zhang^1^, Lei Mu^1^, Yaomin Dai^6^, Fengqi Song^4,5^, Hugen Yan^1^*

*Corresponding author. Email: [chongwang@bit.edu.cn](mailto:chongwang@bit.edu.cn) (C.W.), [feifucong@nju.edu.cn](mailto:feifucong@nju.edu.cn) (F.F.), and [hgyan@fudan.edu.cn](mailto:hgyan@fudan.edu.cn) (H.Y.).

**Supplementary notes:**

**1: Characterization of the Composition of Mo*_x_*W_1-_*_x_*Te_2_.**

**2: Polarization Dependence of LSPRs in Skew Ribbon Arrays.**

**3: The Selection of the Skew Angle** $\boldsymbol{\theta}$**.**

**4: Polarization Dependence of the Drude Response in Skew Ribbon Arrays.**

**5: Fitting Details and Errors of LSPRs in Skew Ribbon Arrays.**

**6: Simulations of Polarized Extinction Spectra in Skew Ribbon Arrays.**

**7: Conductivities Extracted from the Plasmon Dispersion of WTe_2_.**

**8: Ribbon Width Dependence of LSPRs in Mo*_x_*W_1-_*_x_*Te_2_ Skew Ribbons.**

**9: LSPRs in Relatively Large Mo*_x_*W_1-_*_x_*Te_2_ Disks.**

**10: Fitting of the Extinction Spectra of Bare Films and the Extracted Drude Weights.**

**11: IFCs of Plasmon Dispersion in Mo*_x_*W_1-_*_x_*Te_2_.**

**12: Temperature Dependence of the OTT in WTe_2_.**

**1. Characterization of the Composition of Mo*_x_*W_1-_*_x_*Te_2_.**

The final crystal composition was characterized using energy dispersive spectroscopy (EDS) with a scanning electron microscope. EDS measurements were taken at multiple locations on the crystal surface to obtain an average composition. Fig. S1a and b show the typical EDS at 27.8% and 50% Mo doping, respectively.


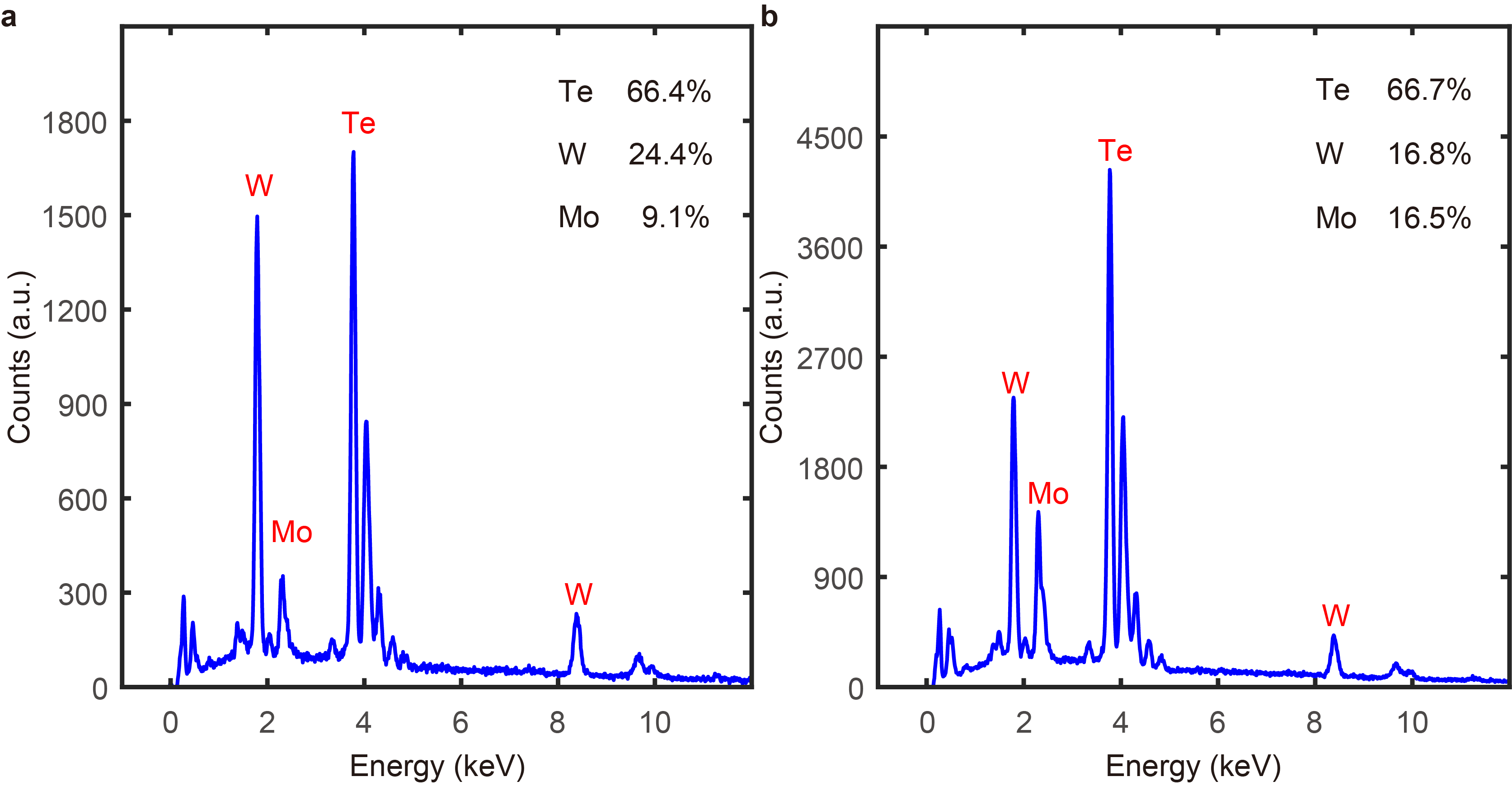


**Fig. S1 | The typical EDS at 27.8% (a) and 50% (b) Mo doping.**

**2. Polarization Dependence of LSPRs in Skew Ribbon Arrays.**

Equation (1) in the main text fully captures the polarization dependence and we derive it in detail now. Here the Ohmic loss (real part of the conductivity) is neglected for convenience. The conclusion, however, is general and rigorous. LSPRs can be treated as self-sustained dipoles, mimicking mechanical oscillators, which give rise to the polarization current density $\boldsymbol{J}_{\mathrm{polar}}$ and the depolarization field $\boldsymbol{E}_{\mathrm{depol}}$. Note that the LSPR in the ribbons is independent of the existence of the external field, which only serves as the external stimuli to start the plasmon dipole oscillation and to compensate the damping afterwards, if there is any. Again, this is fully analogous to a mechanical damped oscillator. $\boldsymbol{J}_{\mathrm{polar}}\cdot\boldsymbol{E}_{\mathrm{ext}}$ accounts for the energy provided by the external field to the plasmon system. Therefore, the most efficient way to drive and feed the plasmon resonance is to let $\boldsymbol{E}_{\mathrm{ext}}$ trace the polarization current density $\boldsymbol{J}_{\mathrm{polar}}$, that is, $\boldsymbol{E}_{\mathrm{ext}}$ is parallel to $\boldsymbol{J}_{\mathrm{polar}}$, as displayed in Fig. S2.

The polarization current density $\boldsymbol{J}_{\mathrm{polar}}$ is equal to $\frac{\partial\boldsymbol{P}}{\partial t}$. Here, ***P*** is the electric polarization caused by the depolarization field (rather than the external field) $\boldsymbol{P}=\chi\varepsilon_{0}\boldsymbol{E}_{\mathrm{depol}}\propto\sigma^{''}\boldsymbol{E}_{\mathrm{depol}}$, where $\chi$ is the polarizability tensor. The polarization $\boldsymbol{P}_{\mathrm{ext}}$ directly generated by the external field $\boldsymbol{E}_{\mathrm{ext}}$ is in-phase with $\boldsymbol{E}_{\mathrm{ext}}$, hence the associated current $\frac{\partial\boldsymbol{P}_{\mathrm{ext}}}{\partial t}$ is out of phase and doesn’t take energy from the external field. Note that $\boldsymbol{E}_{\mathrm{depol}}$ is always perpendicular to the ribbon since polarization charges are along the two edges of the long ribbon, so:

$$\begin{aligned} \boldsymbol{J}_{\mathrm{polar}}\propto\left( \begin{matrix} \sigma_{aa}^{''} & 0 \\ 0 & \sigma_{bb}^{''} \end{matrix} \right)\cdot\left( \begin{matrix} \left| \boldsymbol{E}_{\mathrm{depol}} \right|\cos\theta\\ \left| \boldsymbol{E}_{\mathrm{depol}} \right|\sin\theta\end{matrix} \right)\propto\left( \begin{matrix} \sigma_{aa}^{''}\left( \omega\right)\cos\theta\\ \sigma_{bb}^{''}\left( \omega\right)\sin\theta\end{matrix} \right)\#\left( S1 \right) \end{aligned}$$

where $\sigma_{aa}^{''}$ and $\sigma_{bb}^{''}$ are the imaginary parts of the diagonal elements of the anisotropic conductivity tensor, and $\theta$ is the skew angle of the ribbon array. The polarization of the incident light for the maximal plasmon intensity is defined as $\phi_{\max}$ in the main text. Thus, at such polarization, we have $\boldsymbol{E}_{\mathrm{ext}}=\left( \begin{matrix} \left| \boldsymbol{E}_{\mathrm{ext}} \right|\cos\phi_{\max} \\ \left| \boldsymbol{E}_{\mathrm{ext}} \right|\sin\phi_{\max} \end{matrix} \right)$, which should be parallel to $\boldsymbol{J}_{\mathrm{polar}}$ as discussed above. In conjunction with equation (S1), we arrive at:

$$\begin{aligned} \tan\phi_{\max}\left( \omega\right)=\frac{\sigma_{bb}^{''}\left( \omega\right)}{\sigma_{aa}^{''}\left( \omega\right)}\tan\theta\#\left( S2 \right) \end{aligned}$$

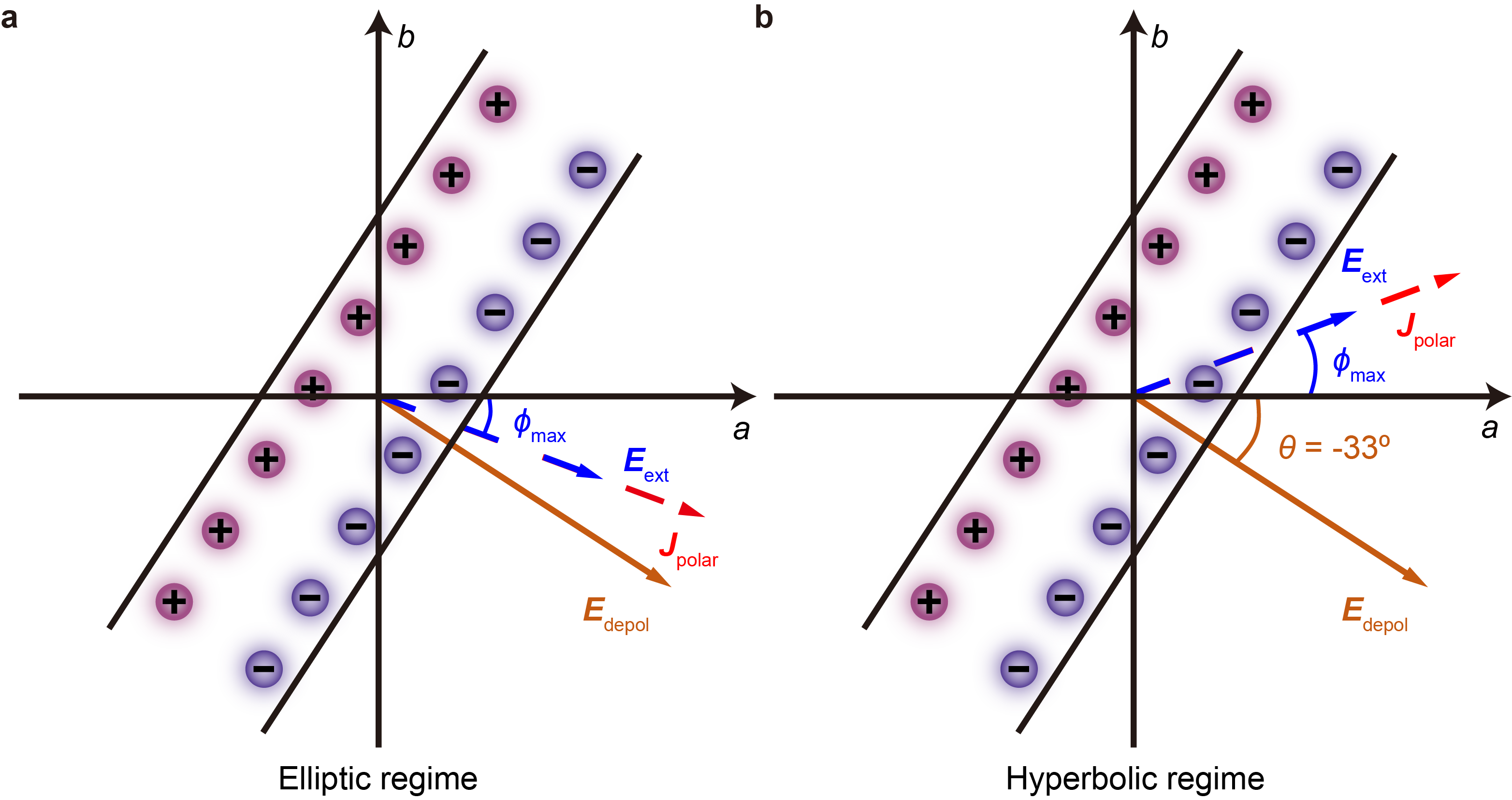


**Fig. S2 | Polarization dependence of plasmon spectra in skew ribbon arrays in the elliptic and hyperbolic regimes respectively.** The optimal polarization for $\boldsymbol{E}_{\mathrm{ext}}$ is indicated.

**3. The Selection of the Skew Angle** $\boldsymbol{\theta}$**.**

A skew angle of -33$^{\circ}$ is chosen based on the following factors: 1), the value of the optimal polarization $\phi_{\max}$ at each resonance frequency $\omega$; 2), the maximal resonance frequency $\omega_{\max}$ we can achieve in the skew ribbon arrays with the skew angle $\theta$. Note that we are discussing the absolute value here because the sign of the skew angle (or the polarization angle $\phi$) depends on our definition arbitrarily and does not affect the conclusion.

Firstly, according to equation (1) or (S2), $\phi_{\max}\left( \omega\right)$ increases with $\theta$, as shown in Fig. S3. Thus, a large $\theta$ is needed to make $\phi_{\max}\left( \omega\right)$ large enough for detection in experiments.

Secondly, $\omega_{\max}$ decreases with the increase of $\theta$. As we know, the ribbon configuration directly determines the wavevector of the plasmon polariton^1^. Specifically, for the ribbon with width $L$, the effective wavevector $\boldsymbol{q}$ is $\pi/L$, and the direction of $\boldsymbol{q}$ is perpendicular to the ribbon. In the elliptic regime, a smaller ribbon width gives a higher plasmon frequency, as the resonance frequency of plasmon polariton typically increases with the wavevector (e.g., the $\omega\propto\sqrt{q}$ relation in the long-wavelength limit). However, in the hyperbolic regime, the dispersion is softened due to the coupling with interband transitions. For example, the maximal resonance frequencies of WTe_2_ along *a-* and *b-*axis are about 632 and 429 cm^-1^, respectively^2^. When the wavevector direction rotates from *a-* to *b*-axis (i.e., when $\theta$ increases), the maximal plasmon frequency decreases. Such effect has been confirmed by our previous work^2^ (Supplementary Figure 9 in Ref. 2). As a result, a smaller $\theta$ can expand the range of the plasmon resonance frequency we can achieve in the skew ribbon arrays.

At last, a moderate skew angle of -33$^{\circ}$ was chosen in our study to ensure a relatively large $\phi_{\max}\left( \omega\right)$ and $\omega_{\max}$ simultaneously.


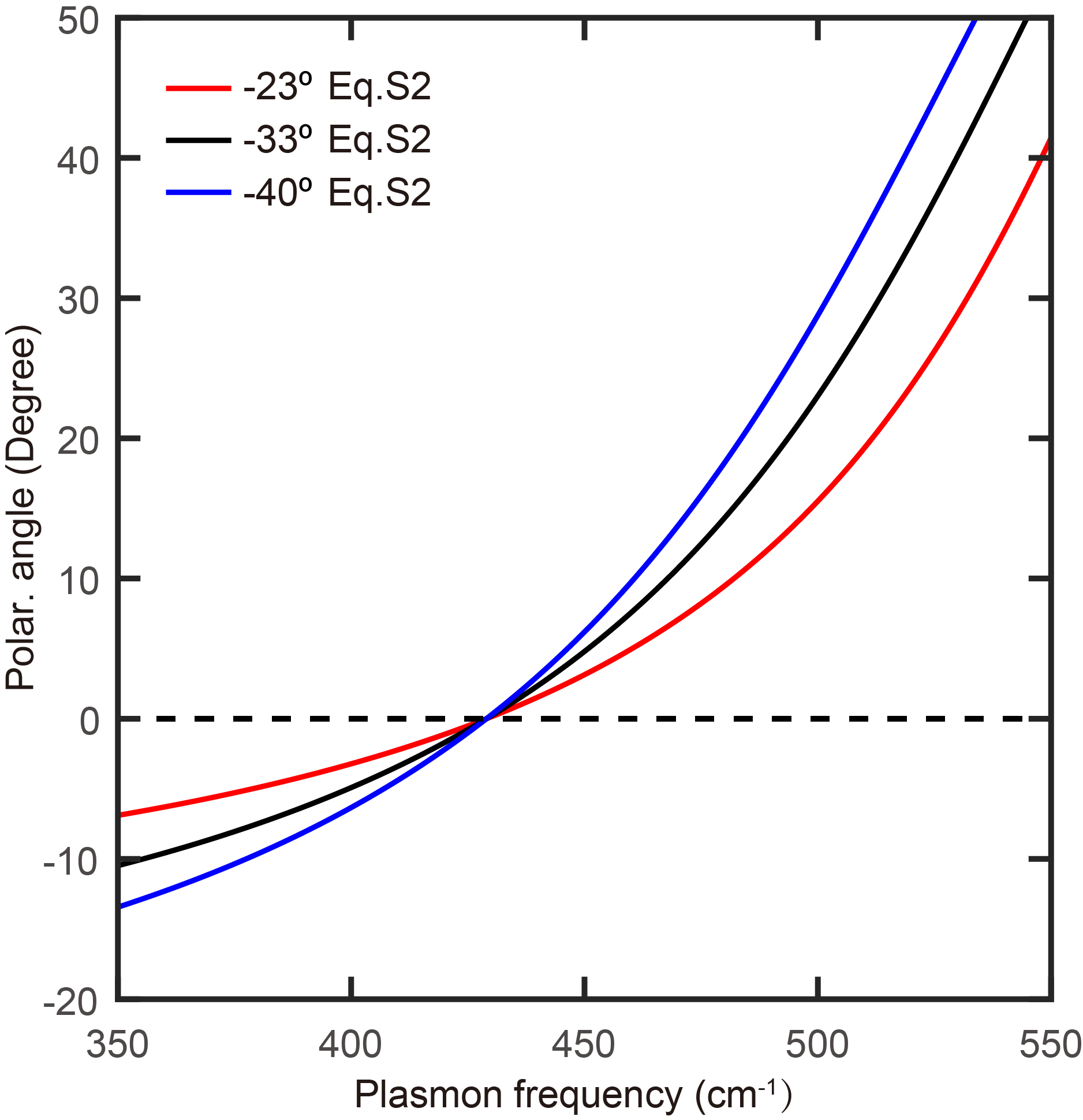


**Fig. S3 | The polarization** $\boldsymbol{\phi}_{\mathbf{max}}$ **as a function of the plasmon frequency at three skew angles (-40**$\boldsymbol{^{\circ}}$**, -33**$\boldsymbol{^{\circ}}$**, -23**$\boldsymbol{^{\circ}}$**).**

**4. Polarization Dependence of the Drude Response in Skew Ribbon Arrays.**

Free carriers are responsible for the Drude response with peak at zero frequency. Fig. S4a exhibits the polarization dependence of the normalized Drude weight of Fig. 2b in the main text. The Drude weight is largest when the polarization is along $57^{\circ}$, which is precisely parallel to the ribbon (skew angle is -33$^{\circ}$). Interestingly, this is the same as that in ribbons made from isotropic two-dimensional films, such as graphene. Here, we’ll give a justification for such a scenario. For simplicity and without loss of generality, the Drude response at zero frequency or the DC response is examined. The light absorption is related to the Joule heating, namely $\boldsymbol{J}\cdot\boldsymbol{E}$, where $\boldsymbol{J}$ and $\boldsymbol{E}$ ($\boldsymbol{E}=\boldsymbol{E}_{\mathrm{ext}}+\boldsymbol{E}_{\mathrm{depol}}$) are the total current density and electric field respectively. The geometrical shape of the ribbon results in the following two features: 1) the depolarization field $\boldsymbol{E}_{\mathrm{depol}}$ is perpendicular to the ribbon due to the translation-invariance of the induced charge distribution at the edges, and 2) the current density component perpendicular to the ribbon edge $J_{\perp}$ must vanish, and only $J_{\parallel}$ survives after it reaches quasi-static state.

We can reach such a conclusion that the polarization for maximal Drude weight is always along the ribbon direction in two ways. The first way is to consider the situation when the external field is perpendicular to the ribbon. As we’ll show, there is no current (either $J_{\perp}$ or $J_{\parallel}$) at all when it reaches the static state and no power is consumed. According to feature 1), the total electric field $\boldsymbol{E}$ is perpendicular to the ribbon as well. Therefore, to meet feature 2), $\boldsymbol{E}$ must be zero to get the zero value of $J_{\perp}$, i.e., the external field and depolarization field compensate each other (equal and opposite). As a result, there is no steady current when the external field is perpendicular to the ribbon, namely $\boldsymbol{J}=0$ and no Joule heating (Drude response) at all. Since we have found the minimum, the maximal Drude response occurs at the polarization perpendicular to the minimal case, i.e., parallel to the ribbon.

The second way is to directly find the maximum for $\boldsymbol{J}\cdot\boldsymbol{E}$ at different polarization with constant external field amplitude $\left| \boldsymbol{E}_{\mathrm{ext}} \right|$. As mentioned above (feature 1), $\boldsymbol{J}$ has a definitive direction which is parallel to the ribbon. According to the Ohm’s law $\boldsymbol{J}=\sigma\boldsymbol{E}$, where $\sigma$ is the conductivity tensor,

$$\begin{aligned} \boldsymbol{J}=\left( \begin{matrix} \left| \boldsymbol{J} \right|\cos\alpha\\ \left| \boldsymbol{J} \right|\sin\alpha\end{matrix} \right)=\sigma\boldsymbol{E}=\left( \begin{matrix} \sigma_{aa} & 0 \\ 0 & \sigma_{bb} \end{matrix} \right)\left( \begin{matrix} \left| \boldsymbol{E} \right| \cos\beta\\ \left| \boldsymbol{E} \right| \sin\beta\end{matrix} \right)=\left( \begin{matrix} {\left| \boldsymbol{E} \right|\sigma}_{aa}\cos\beta\\ \left| \boldsymbol{E} \right|\sigma_{bb} \sin\beta\end{matrix} \right)\boldsymbol{\#}\left( S3 \right) \end{aligned}$$

where $\sigma_{aa}$ and $\sigma_{bb}$ are the diagonal elements of the conductivity tensor (real parts), $\alpha$ and $\beta$ are the tilted angles of the ribbon and the total electric field $\boldsymbol{E}$ respectively, as shown in Fig. S4b. Therefore, for a given conductivity tensor, the total electric field $\boldsymbol{E}$ has a fixed orientation $\beta$:

$$\begin{aligned} \tan\beta=\frac{\sigma_{aa}}{\sigma_{bb}}\tan\alpha\#\left( S4 \right) \end{aligned}$$

Now the Drude response is:

$$\begin{aligned} \boldsymbol{J}\cdot\boldsymbol{E}=\left( \begin{matrix} {\left| \boldsymbol{E} \right|\sigma}_{aa}\cos\beta& \left| \boldsymbol{E} \right|\sigma_{bb} \sin\beta\end{matrix} \right)\cdot\left( \begin{matrix} \left| \boldsymbol{E} \right| \cos\beta\\ \left| \boldsymbol{E} \right| \sin\beta\end{matrix} \right)=\left| \boldsymbol{E} \right|^{2}\left( \sigma_{aa}{\cos\beta}^{2}+\sigma_{bb}{\sin\beta}^{2} \right)\boldsymbol{\#}\left( S5 \right) \end{aligned}$$

Obviously, the maximum of $\left| \boldsymbol{E} \right|$ leads to the maximal absorption, since $\beta$ is fixed according to equation (S4). Because $\boldsymbol{E}$ has a fixed direction and one of its components $\boldsymbol{E}_{\mathrm{depol}}$ is always perpendicular to the ribbon, we can find its maximum geometrically. We draw a circle with radius $\left| \boldsymbol{E}_{\mathrm{ext}} \right|$ (blue dashed circle), as shown in Fig. S4b. According to the vector summation rule (triangle rule) for $\boldsymbol{E}=\boldsymbol{E}_{\mathrm{ext}}+\boldsymbol{E}_{\mathrm{depol}}$, the external field $\boldsymbol{E}_{\mathrm{ext}}$ parallel to the ribbon gives the maximal $\left| \boldsymbol{E} \right|$. Therefore, the Drude response is largest when the external field is applied along the ribbon direction. This rationalizes the experimental finding in Fig. S4a.


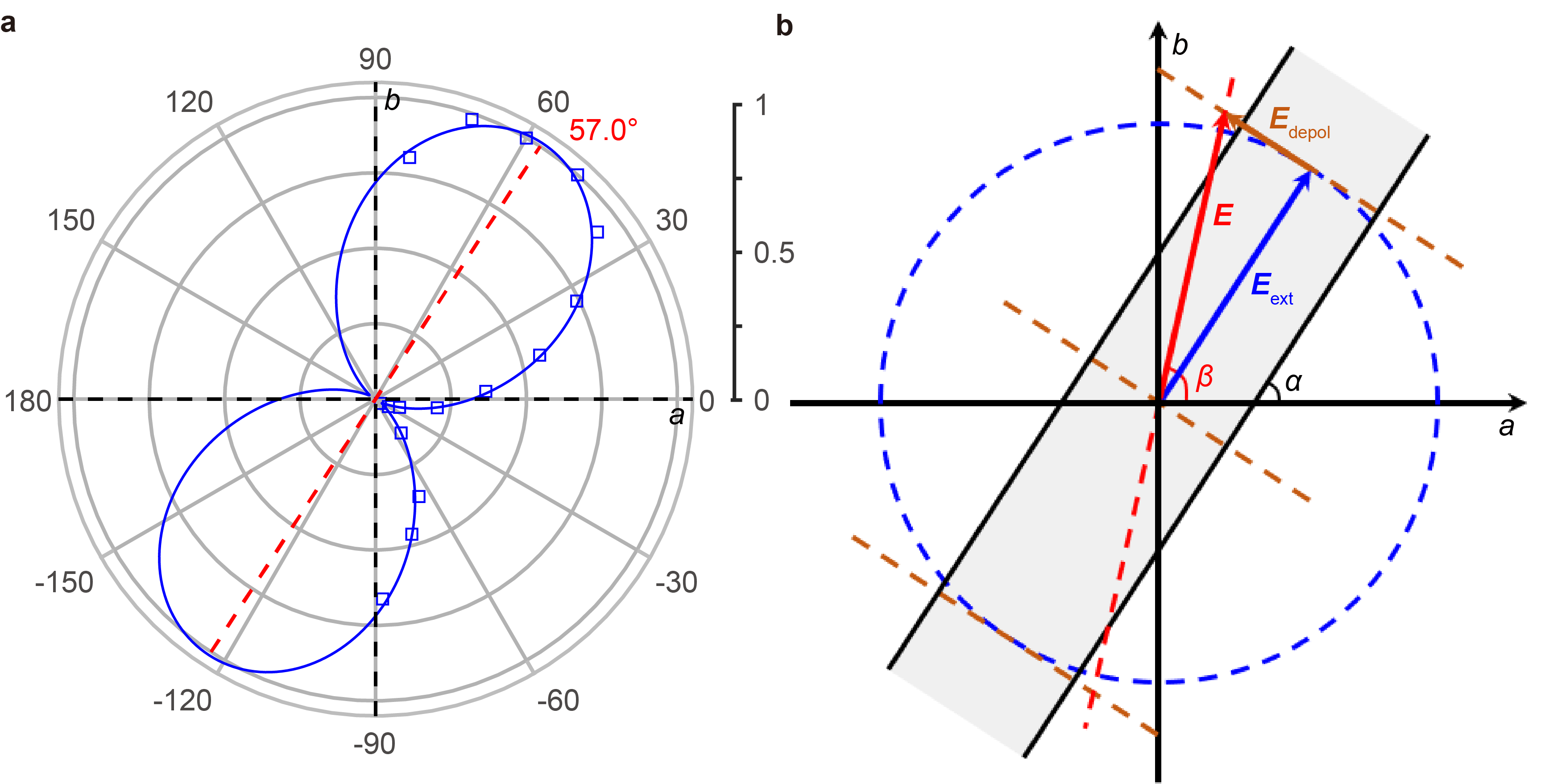


**Fig. S4 | Polarization dependence of the Drude response in skew ribbon arrays. a**, Polarization dependence of the normalized Drude weight of Fig. 2b in the main text. Black dashed lines denote the crystal axes. The parallel direction to ribbons is labeled by the red dashed line with tilted angle of $57^{\circ}$, which is also the light polarization for the maximal Drude weight. **b**, A sketch of electrical fields in a ribbon. The depolarization field $\boldsymbol{E}_{\mathrm{depol}}$ is perpendicular to the ribbon edge. The tilted angles of the ribbon and the total electric field $\boldsymbol{E}$ are $\alpha=57^{\circ}$ and $\beta$, respectively.

**5. Fitting Details and Errors of LSPRs in Skew Ribbon Arrays.**

The fitting details of the spectra in Fig. 2a in the main text are plotted in Fig. S5 and listed in Table S1. The Drude weight *D*, the plasmon intensity $S_{p}$ and the interband transition intensity $S_{i}$ are fitting parameters. The plasmon frequency, the interband transition frequency, the Drude and plasmon scattering rates are fixed at 308, 800, 60 and 90 cm^-1^ respectively.

In our experiments, the main sources of error are associated with two parameters: the plasmon resonance frequency $\omega$ and the optimal polarization angle $\phi_{\max}\left( \omega\right)$. To determine the error for the first parameter, the raw polarized spectra were fitted using different initial fitting parameters to calculate the average of the resonance frequency. As for $\phi_{\max}$, the error can be attributed to two factors. Firstly, errors arise from the fabrication, such as cutting the skew angle of -33$^{\circ}$, and the spectrum acquiring process, such as measuring the polarization angle $\phi$ (with errors of approximately 1$^{\circ}$). Secondly, the fitting procedure, which involves fitting the polarization dependence of the plasmon spectral weight by $\cos^{2} \phi$, introduces a principal error in $\phi_{\max}$ of about 1.5$^{\circ}$. To obtain an overall estimation of the error in $\phi_{\max}$, the contributions from these two factors were combined using the error transfer formula, as shown in Fig. 3a-c in the main text.


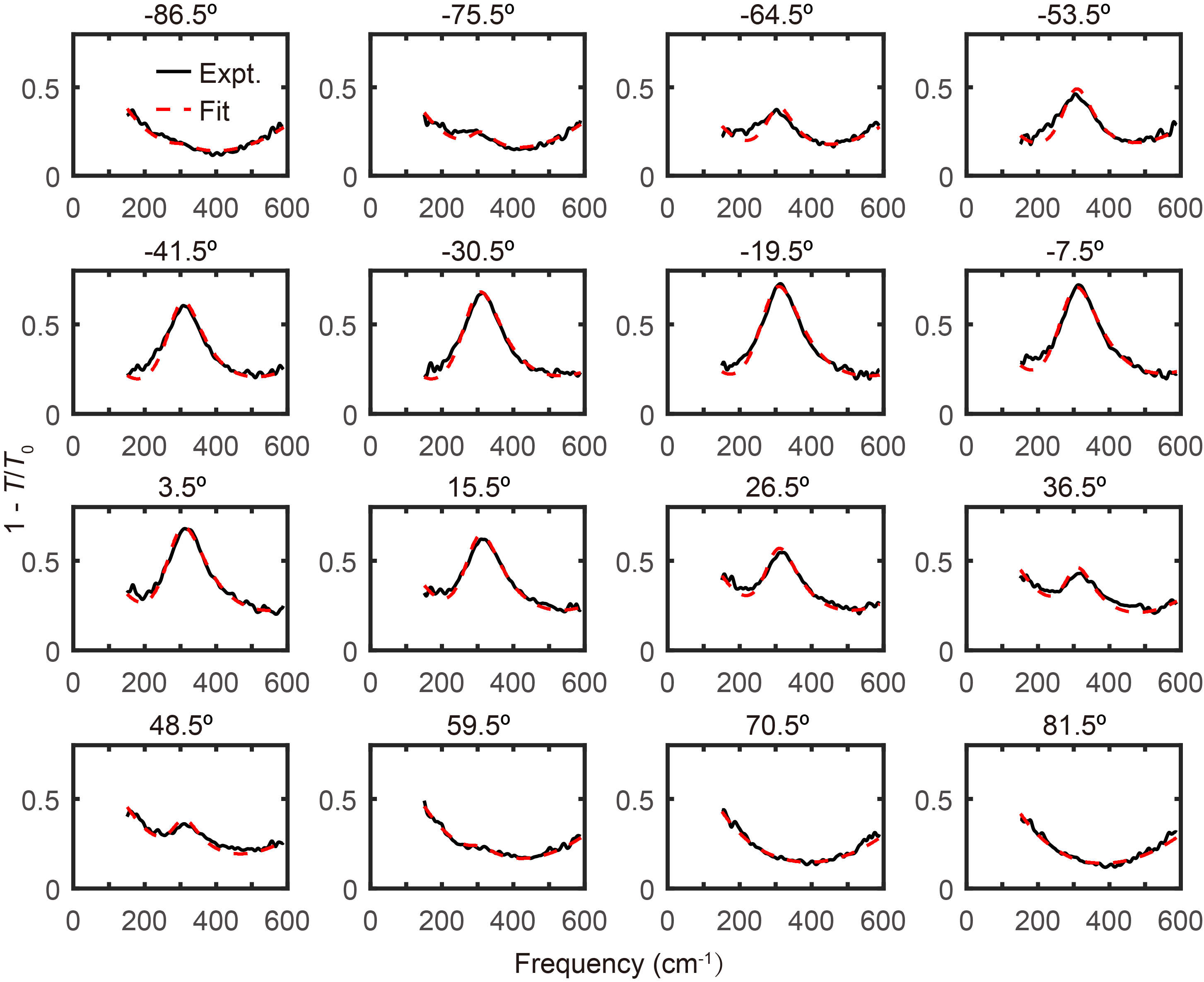


**Fig. S5 | The experimental (solid lines) and fitted (dashed lines) extinction spectra at different polarizations (from -86.5**$\boldsymbol{^{\circ}}$ **to 81.5**$\boldsymbol{^{\circ}}$**) from Fig. 2a in the main text.**

| Polar. angle ($^{\circ}$) | *D* (${10}^{18}$ a.u.) | $S_{p}$ (${10}^{17}$ a.u.) | $S_{i}$ (${10}^{18}$ a.u.) |
| --- | --- | --- | --- |
| -86.5 | 1.49 | 0.32 | 2.87 |
| -75.5 | 1.40 | 1.20 | 3.09 |
| -64.5 | 1.08 | 3.49 | 2.83 |
| -53.5 | 0.86 | 5.71 | 2.50 |
| -41.5 | 0.79 | 9.55 | 2.07 |
| -30.5 | 0.72 | 11.8 | 1.80 |
| -19.5 | 0.86 | 13.2 | 1.41 |
| -7.5 | 1.03 | 12.8 | 1.69 |
| 3.5 | 1.24 | 11.7 | 1.60 |
| 15.5 | 1.46 | 9.72 | 1.83 |
| 26.5 | 1.71 | 6.97 | 2.24 |
| 36.5 | 1.86 | 4.19 | 2.64 |
| 48.5 | 1.87 | 2.76 | 2.55 |
| 59.5 | 1.87 | 0.58 | 2.96 |
| 70.5 | 1.72 | 0.02 | 2.99 |
| 81.5 | 1.67 | 0.07 | 3.04 |

**Table S1 | Fitting parameters of the spectra in Fig. 2a in the main text.**

**6. Simulation of Polarized Extinction Spectra in Skew Ribbon Arrays**

We simulated the spectra in WTe_2_ skew ribbon arrays of the skew angle $\theta=-33^{\circ}$ to verify the equation (1) or (S2). The extinction spectra (denoted as 1-*T*) of five different suspended skew ribbon arrays, with ribbon widths of 0.6, 0.8, 2, 3, 4 $\mu m$ (different wavevectors) respectively, were calculated using the finite-element software (Comsol Multiphysics) with a refined tetrahedral mesh and the frequency-domain solver of Maxwell equations. The complex dispersive conductivity of WTe_2_ films (treated as a two-dimensional layer) was determined by previous results^2^ (also the following Note 7).

Fig. S6 displays simulations of three representative samples, with resonance frequencies in the elliptic regime (394 cm^-1^), the vicinity of the topological transition point (439 cm^-1^) and the hyperbolic regime (499 cm^-1^), respectively. The corresponding extinction spectra with different incident light polarization (from -90$^{\circ}$ to 90$^{\circ}$, with intervals of 11.25$^{\circ}$) are plotted as pseudo color maps in Fig. S6a-c. The angle for maximal plasmon intensity gradually evolves from negative to positive with incremental resonance frequency, demonstrating the clear evidence for the topological transition. Moreover, the Drude response is most intense when the polarization is along the ribbon (57$^{\circ}$). These spectra were further fitted by the Drude-Lorentz model to extract the plasmon intensity. The absorption spectra due to the plasmon polariton are plotted in Fig. S6d-f. The extracted plasmon intensity was fitted as a function of $\cos^{2} \phi$ ($\phi$ is the polarization angle) as shown in Fig. S6g-i. Consistent with equation (1) or (S2), the polarization angle for the maximal plasmon intensity $\phi_{\max}$ is negative (positive) for the elliptic (hyperbolic) topology of the plasmon dispersion and is approximately zero near the transition point. The fitted $\phi_{\max}$ from simulations and the calculated $\phi_{\max}$ by equation (S2) with the corresponding conductivity are listed in Table S2 for quantitative comparison, which agree well with each other (errors originate from the fitting of the simulated spectra). In summary, the simulations verify the theory (Note 2) convincingly.


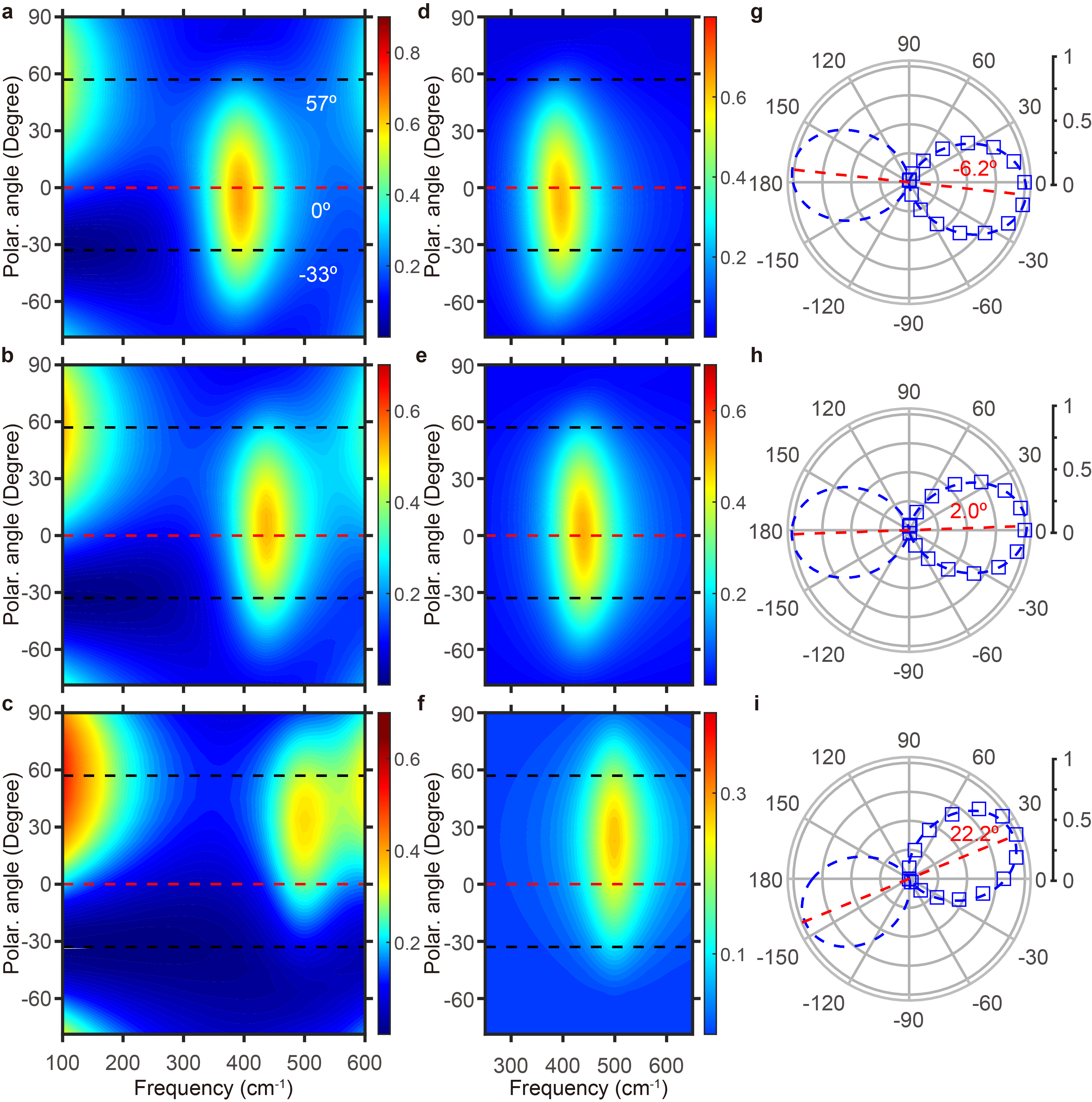


**Fig. S6 | The simulated polarization dependence of plasmon spectra in skew ribbon arrays.** **a-c,** Polarization dependence of simulated extinction spectra at plasmon frequencies of 394, 439, 499 cm^-1^, respectively. **d-f,** The corresponding pseudo color maps of the absorption spectra due to the plasmon polariton in **a-c**. **g-i,** Polarization dependence of the corresponding normalized plasmon weights of **a-c** or **d-f**. In **a-f**, black dashed lines denote the parallel (57$^{\circ}$) and perpendicular (-33$^{\circ}$) directions with respect to the ribbon respectively, and the red dashed lines denote *a*-axis.

| Plasmon frequency (cm^-1^) | $\phi_{\max}$ ($^{\circ}$) by simulations | $\phi_{\max}$ ($^{\circ}$) by equation (S2) |
| --- | --- | --- |
| 354 | -10.5$\pm$0.6 | -10.1 |
| 394 | -6.2$\pm$0.5 | -5.7 |
| 439 | 2.0$\pm$0.5 | 2.1 |
| 499 | 22.2$\pm$0.6 | 22.5 |
| 507 | 26.7$\pm$0.7 | 26.6 |

**Table. S2 | The** $\boldsymbol{\phi}_{\mathbf{max}}$ **determined by simulations and calculations of equation (S2).**

**7. Conductivities Extracted from the Plasmon Dispersion of WTe_2_.**

The detailed fitting process to obtain the optical conductivity of the WTe_2_ film from the plasmon dispersion has been discussed in the main text of Ref. 2. Here we briefly reiterate. The plasmon resonance frequencies along principal crystal axes were measured in WTe_2_ rectangular arrays, and the corresponding wavevectors were determined by the structure size. The loss function -Im($1/\varepsilon$), defined by the imaginary part of the inverse of the dielectric function, was calculated to fit the plasmon dispersion. For the two-dimensional case, the non-local dielectric function is defined as follows,

$$\begin{aligned} \varepsilon=\varepsilon_{\mathrm{env}}+\frac{i\sigma\left( \omega\right)}{\varepsilon_{0}\omega}\cdot\frac{q}{2}\#\left( S6 \right) \end{aligned}$$

where $\varepsilon_{\mathrm{env}}$ is the substrate permittivity and $q$ is the wavevector (since we consider two principal axes, the vector form of $q$ and tensor form of $\sigma$ are omitted). The conductivities along two principal axes, which are assumed to be a Drude term plus a Lorentz term with parameters to be determined, were obtained by fitting the anisotropic plasmon dispersion using the loss function -Im($1/\varepsilon$) and equation (S6). The obtained optical conductivities were then substituted into equation (1) (or (S2)) to draw the black solid line in Fig. 3a in the main text.

**8. Ribbon Width Dependence of LSPRs in Mo*_x_*W_1-_*_x_*Te_2_ Skew Ribbons.**

In order to observe LSPRs across different frequencies and track the OTT, we changed the ribbon width. Fig. S7 displays the plasmon dispersion of Mo*_x_*W_1-_*_x_*Te_2_ (*x* = 0.278) acquired from skew ribbon arrays ($\theta=-33^{\circ}$) of different width. The thickness *t* of the exfoliated film of Mo*_x_*W_1-_*_x_*Te_2_ in our paper ranges from 40 to 120 nm. In this thickness range, the electronic band structure remains the same as that of the bulk. The wavevector $q$ in a ribbon array^1^ is determined by the ribbon width *L* (usually from 0.3 to 10 $\mu m$) with $q=\pi/L$, which is further normalized to 100 nm thick sample by multiplying $t/{100}$, since the two-dimensional conductivity is proportional to the film thickness *t*. As shown in Fig. S7, only at low frequencies, the plasmon dispersion follows the $\sqrt{q}$relation, a characteristic feature for the two-dimensional plasmon polaritons from free carriers^3^. At higher frequencies, the dispersion softens and departs from the $\sqrt{q}$ relation, which is primarily due to the coupling to interband transitions (fitted by $\left( q/\left( \varepsilon_{\mathrm{env}}+\rho_{0}q \right) \right)^{1/2}$, with $\rho_{0}$ as a parameter for the screening length )^4,5^.


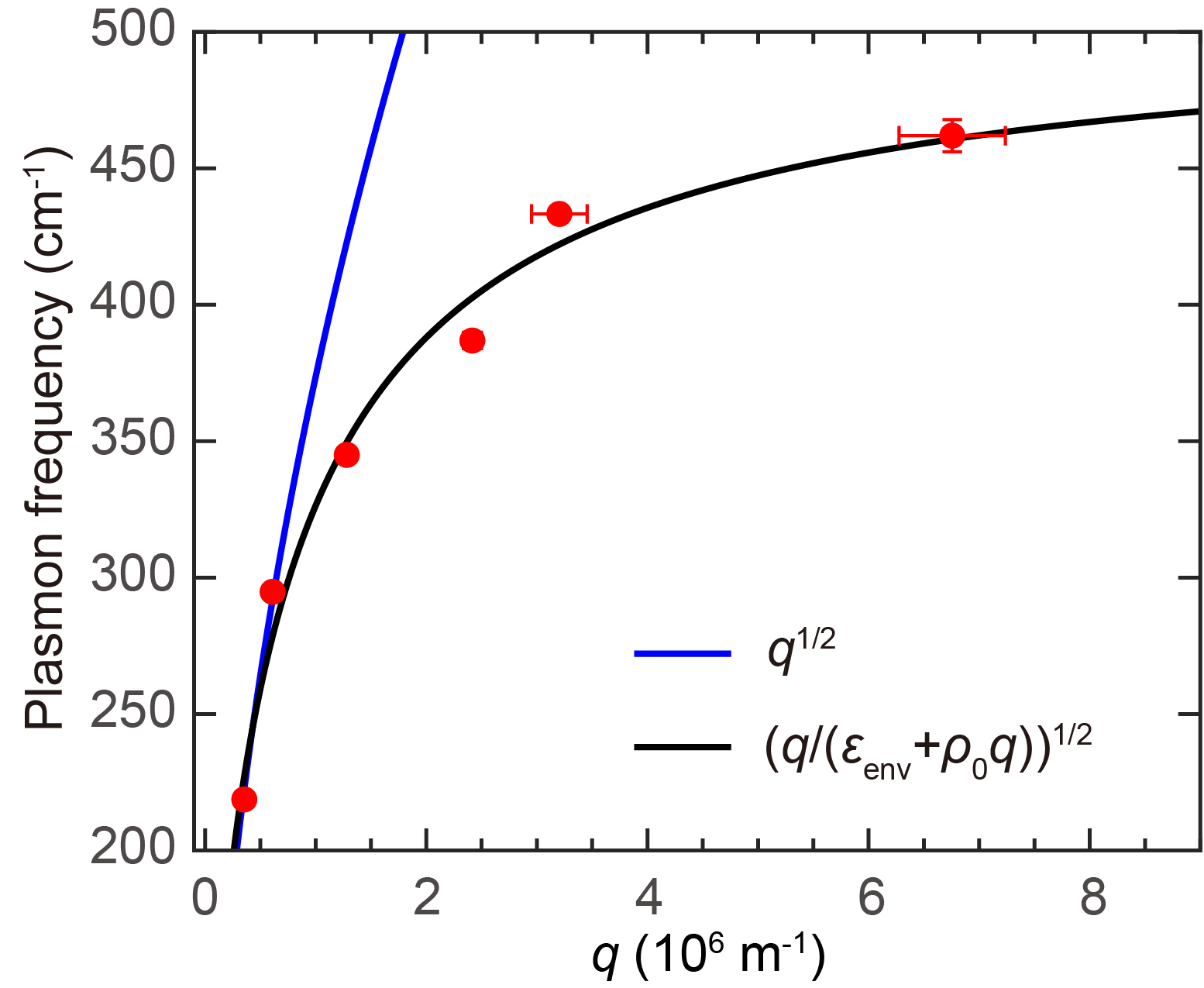


**Fig. S7 | Plasmon dispersion in skew ribbon arrays of Mo*_x_*W_1-_*_x_*Te_2_ (*x* = 0.278).**

**9. LSPRs in Relatively Large Mo*_x_*W_1-_*_x_*Te_2_ Disks.**

To investigate the intrinsic intraband plasmon in Mo*_x_*W_1-_*_x_*Te_2_, disk arrays with larger diameters (lower wavevectors) were patterned, in which the plasmon frequencies are low enough to stay away from the interband transitions, and the dispersion follows the $\sqrt{q}$ relation. The thickness, disk diameter, plasmon frequencies along *a*- and *b*-axis at different doping are summarized in Table S3 for the samples. The extinction spectra and fitting curves are plotted in Fig. S8. LSPRs along *a*-axis exhibit similar FWHMs for different doping levels, which are 86.6, 93, 102.7 cm^-1^ respectively. The quality factors of those LSPRs are 3.4, 3.1, 2.1 respectively. This suggests that the Drude scattering rates are similar for different doping levels and the doping procedure doesn’t degrade the sample quality. Meanwhile, the peak positions of plasmon spectra in Fig. S8 can directly provide us information on the Drude weights, which will be used in Note 10.

| Doping *x* of the sample | 0 | 0.278 | 0.5 |
| --- | --- | --- | --- |
| Thickness (nm) | 100 | 50 | 45 |
| Disk diameter ($\text{μ}$m) | 4 | 2.92 | 4.6 |
| Plasmon frequency along *a*-axis (cm^-1^) | 295.0 | 291.7 | 214.3 |
| Plasmon frequency along *b*-axis (cm^-1^) | 200.7 | 179.0 | 138.3 |

**Table S3 | Sample parameters of disk arrays at different doping.**


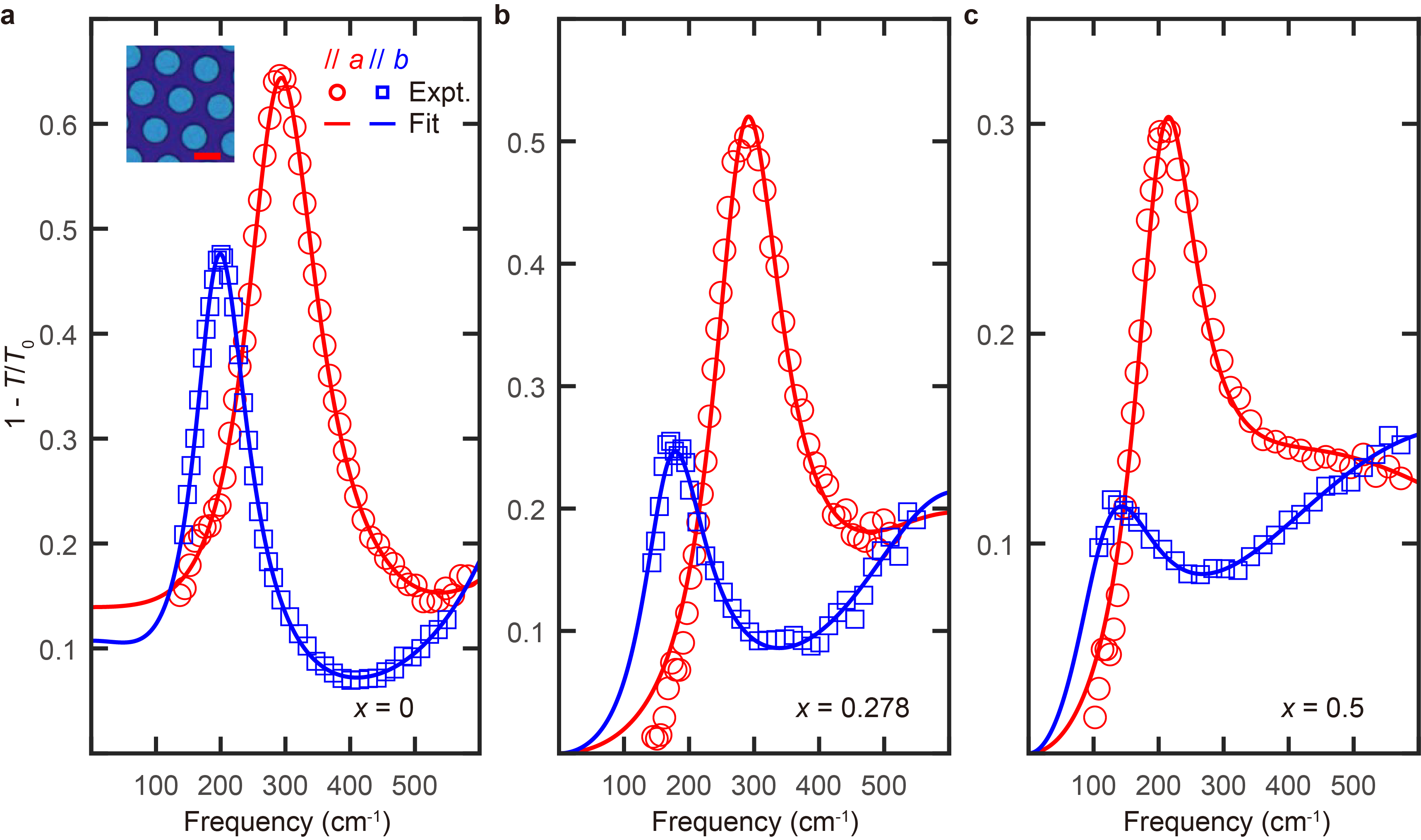


**Fig. S8 | Anisotropic LSPRs of Mo*_x_*W_1-_*_x_*Te_2_ (*x* = 0, 0.278, 0.5) disk arrays.** The upturn of the spectra at low frequency in **a** is due to the Drude absorption from the unpatterned film in the vicinity of the disk array. Inset of **a** shows the optical microscopic image of a disk array at *x* = 0. Scale bar in red is 5$\mu m$.

**10. Fitting of the Extinction Spectra of Bare Films and the Extracted Drude Weights.**

We fit the extinction spectra of bare films using equations (2) and (3) in the main text. The fitting components in the mid- and far-IR regimes are displayed in Fig. S9, where the first low energy interband transition resonances mentioned in the main text in Fig. 4a-c are labelled by ‘L1’ in blue. Here, one Drude component (labeled by ‘D’) and five Lorentz components (labeled by ‘L’) for *a*-axis (seven Lorentz components for *b*-axis) are included. During the fitting, the Drude scattering rate was fixed at the value derived from the corresponding peak width of the low frequency LSPR.

The Drude response in the lower THz regime is beyond our measurement range, leading to possible underestimation of the Drude weights from the fitting at larger doping (particularly, the 50% doping case). For improvements, at doping composition of *x* = 0.5, we resorted to the plasmon spectra in Note 9, since the Drude feature dictates the plasmon feature, which shifts into our measurement range. The plasmon frequency in the small $q$ limit ($\omega\propto\left( q\cdot\frac{n_{e}}{m_{e}} \right)^{1/2}$, $n_{e}$ is the sheet carrier density) at different doping is related to the Drude weight^3^ ($D=\frac{\pi e^{2}n_{e}}{m_{e}}$). Therefore, at known thickness and plasmon wavevector, $D_{0.5}$ (0.5 is the composition ratio) can be calculated according to the peak frequencies of Fig. S8. Specifically, by comparing to the fitted $D_{0}$ of WTe_2_, the Drude weight $D_{0.5}$ can be obtained. By equation (3), the DC conductivity $\sigma_{0.5}\left( 0 \right)$ is equal to $\frac{D_{0.5}}{\pi\Gamma}$, which determines the extinction $A_{0.5}$ at zero frequency according to equation (2). It was included to fit the extinction spectrum of the unpatterned film with *x* = 0.5. Finally, the fitted thickness-normalized Drude weights ($D$ divided by the thickness *t*, namely, the Drude weight for the bulk) for *x* = 0.5 and those obtained through direct fitting of the film spectra for *x* = 0, and *x* = 0.278 are displayed in Fig. S10.


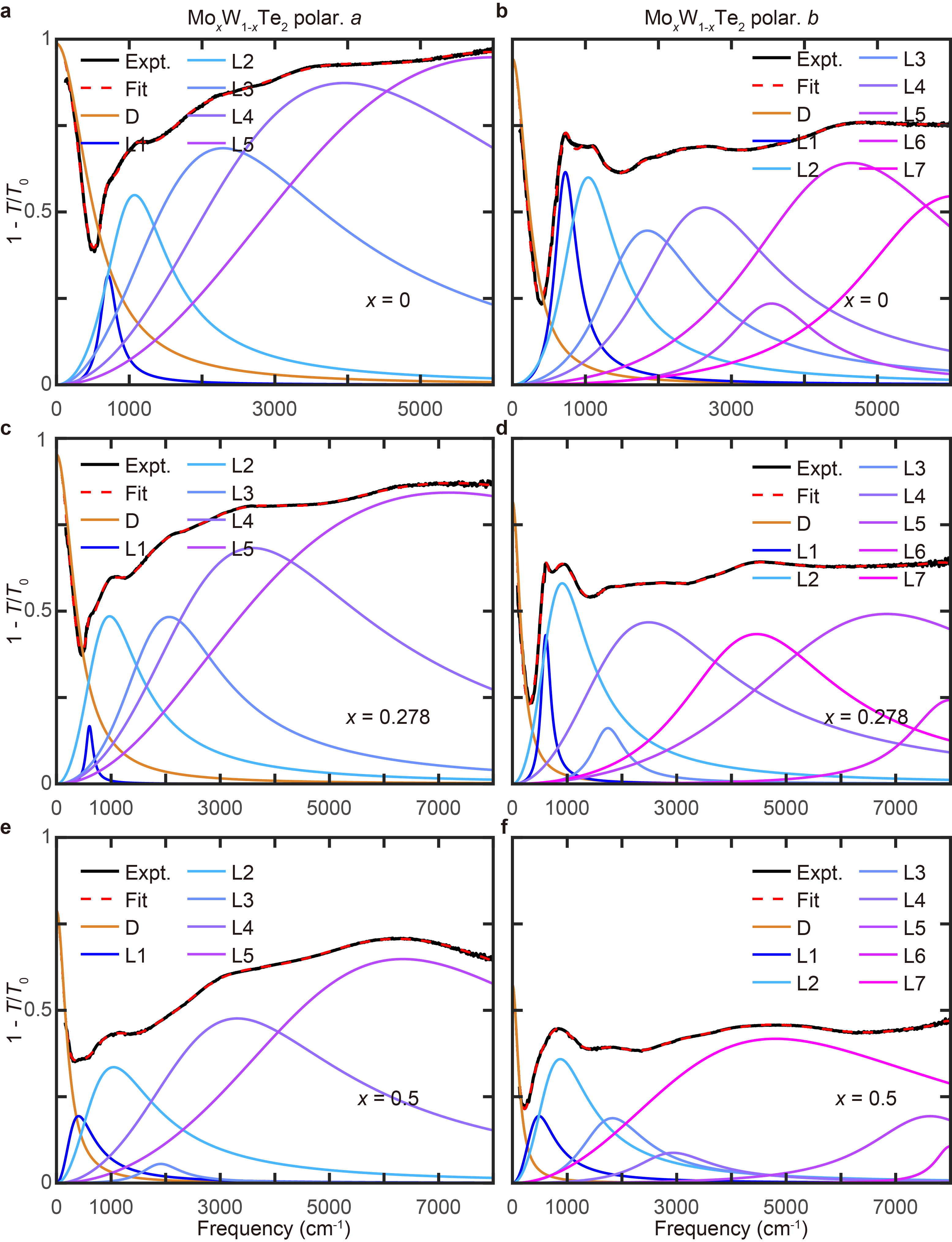


**Fig. S9 | Drude-Lorentz model fittings of extinction spectra in Figs. 4a-c in the main text (here with extended frequency range).** The left and right panels are for *a*-axis and *b*-axis polarizations respectively. The colored solid lines labelled by “D” (Drude response) or “L” (interband transitions) are Drude and Lorentz components, which were obtained by substituting the corresponding conductivity components into equation (2) in the main text.


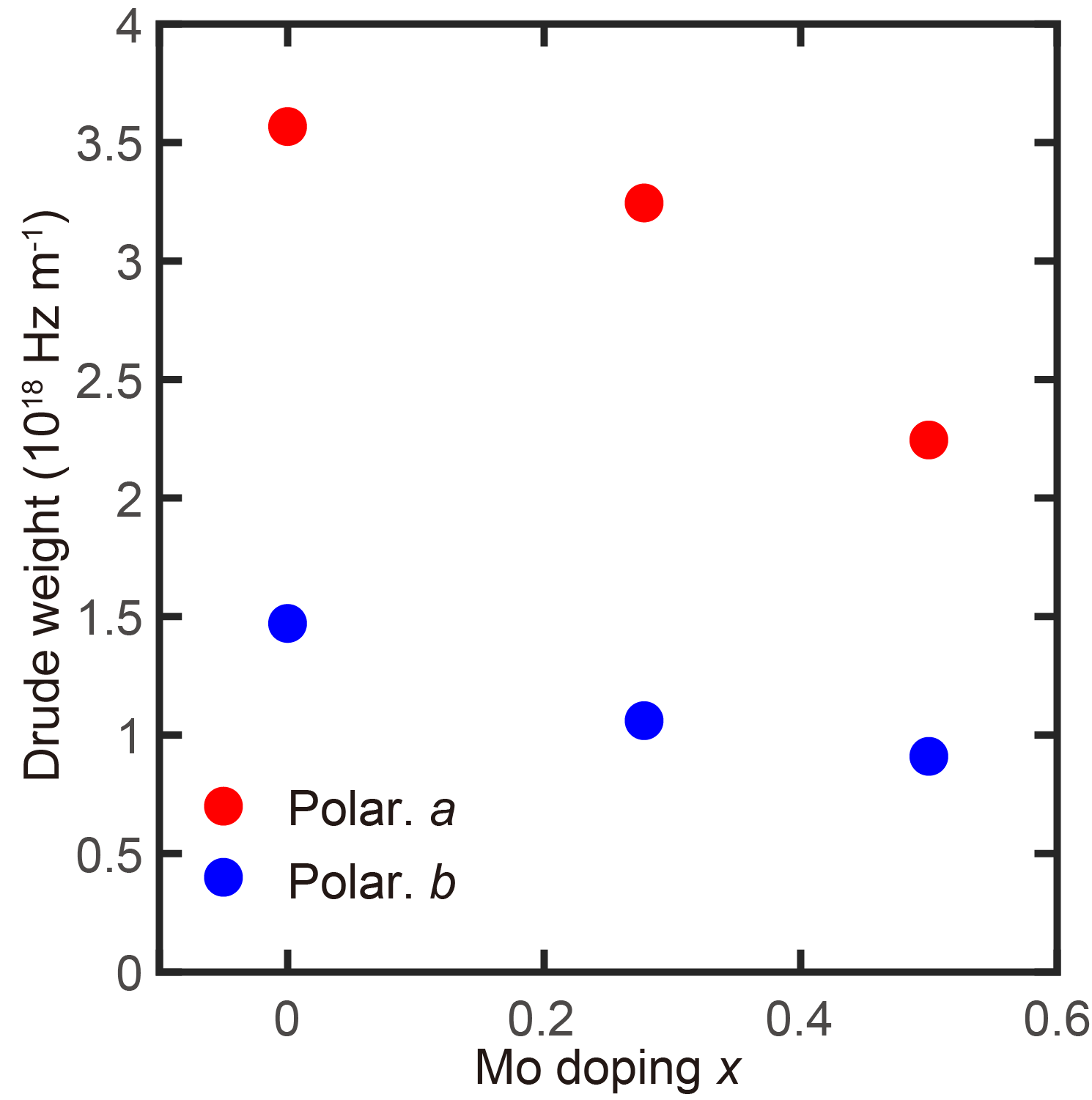


**Fig. S10 | Extracted Drude weights of Mo*_x_*W_1-_*_x_*Te_2_ (*x* = 0, 0.278, 0.5) films from Fig. S9, normalized by the thickness.**

**11: IFCs of Plasmon Dispersion in Mo*_x_*W_1-_*_x_*Te_2_.**

The loss function Im$\left( -1/\varepsilon\right)$ (the imaginary part of the inverse of the dielectric function) is calculated in the two-dimensional wavevector space to plot the IFCs of the plasmon dispersion in WTe_2_ at different Mo doping levels and temperatures. This is achieved by utilizing equation (S6) and the fitted optical sheet conductivity obtained from experimental data (e.g., Fig. 4d-f and Fig. S13b).

Fig. S11 presents pseudocolor maps of the calculated loss function, showcasing the evolution of the IFCs in Mo*_x_*W_1-_*_x_*Te_2_ (a for WTe_2_, b for 27.8% Mo doping, c for 50% Mo doping). The solid black curves in Fig. S11 depict the fitted dispersion (IFCs). In particular, Fig. S11a illustrates the gradual transition of the IFCs from an elliptic shape to a hyperbolic shape as the plasmon resonance frequency increases. The evolution signifies the OTT of the IFCs of plasmon polaritons in WTe_2_. Additionally, the redshift of the OTT energy with increasing Mo doping is evident, which aligns with the findings from our polarization experiments.

Using the same method, the temperature dependence of the IFCs at a specific frequency (440 cm^-1^) in WTe_2_ is also examined, as depicted in Fig. S12. As the temperature increases, the IFCs undergo a gradual transition from a hyperbolic shape to an elliptic shape. This observation is in line with the blueshift of the energy of the OTT at higher temperatures, as illustrated in Fig. S13b.


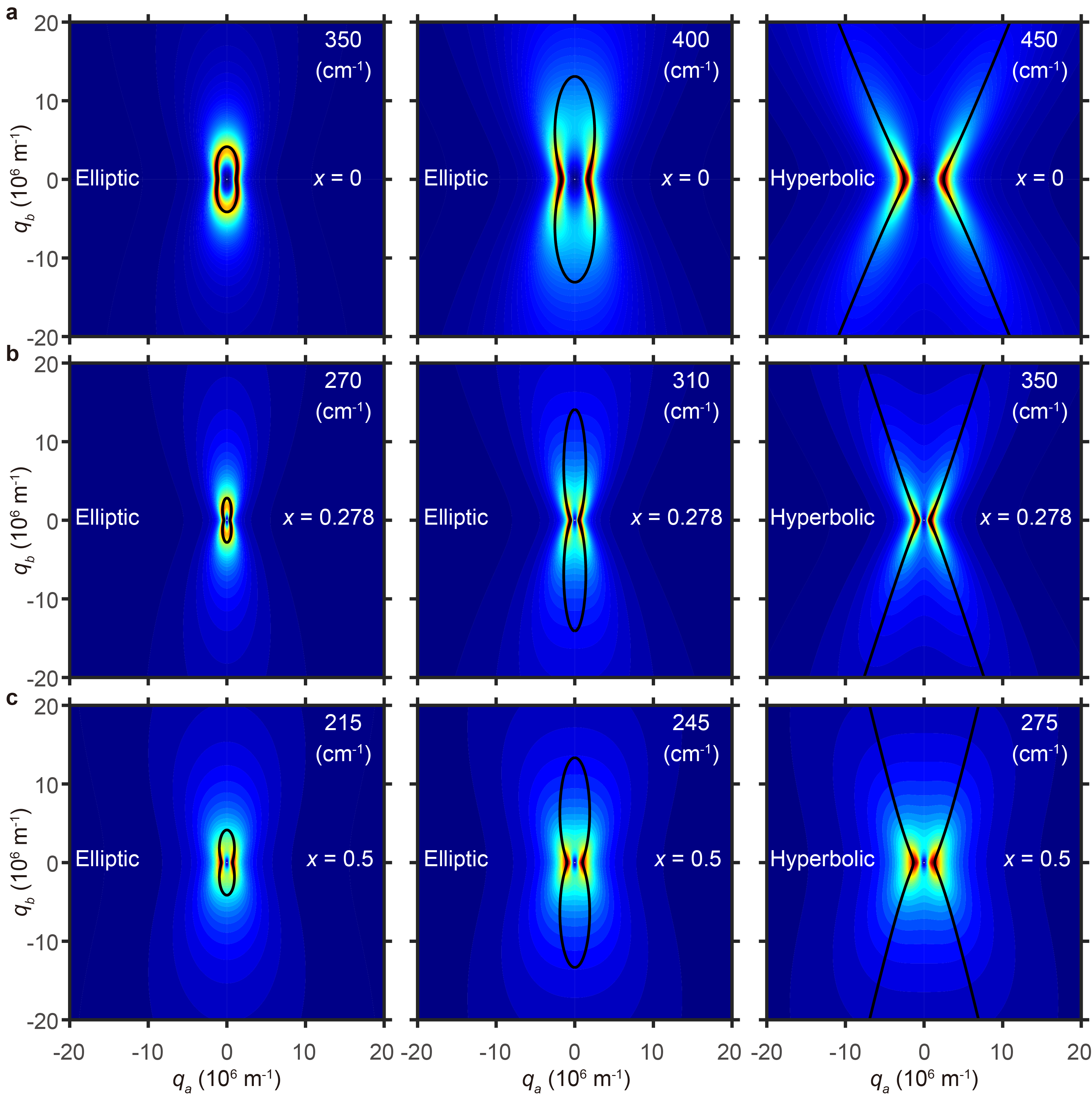


**Fig. S11 | Mo doping effect on the IFCs of plasmon dispersion at different resonance frequencies in WTe_2_.** The Mo doping *x* is 0, 0.278, 0.5 in **a**, **b** and **c** respectively. The loss function is displayed as pseudocolor maps in the two-dimensional wavevector space. The solid black curves represent the fitted dispersion (IFCs) of plasmon polaritons.


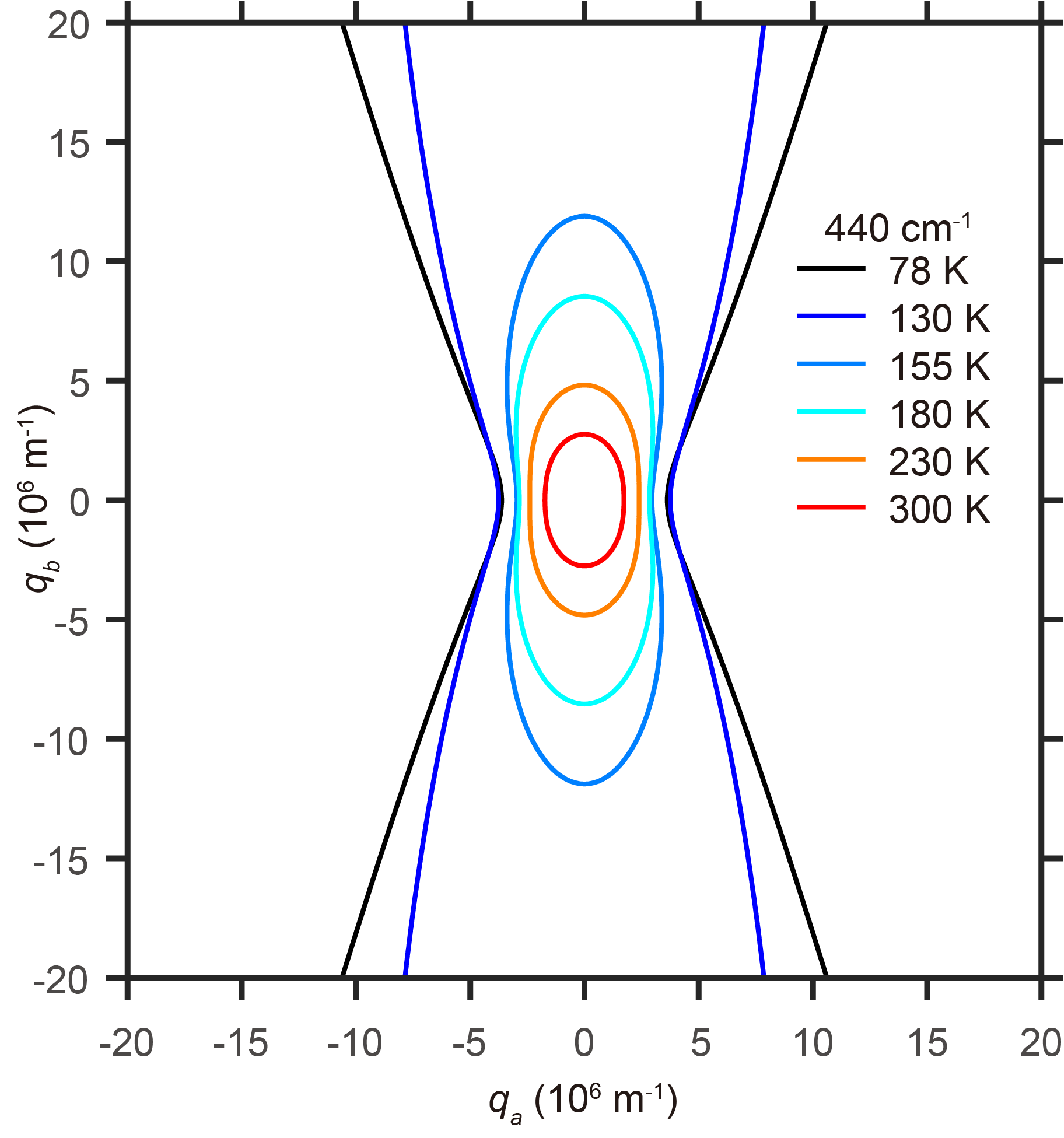


**Fig. S12 | Temperature dependence of the IFCs of plasmon dispersion in WTe_2_.** The solid curves are fitted to the calculated loss functions at each temperature. The plasmon resonance frequency is 440 cm^-1^.

**12. Temperature Dependence of the OTT in WTe_2_.**

The extinction spectra and their fitting curves (here with extended frequency range) of a WTe_2_ bare film (35 nm thickness), the extracted imaginary parts of the optical conductivities along both axes, and the polarization angle $\phi_{\max}$ as a function of the plasmon frequency at different temperatures are displayed in Fig. S13.


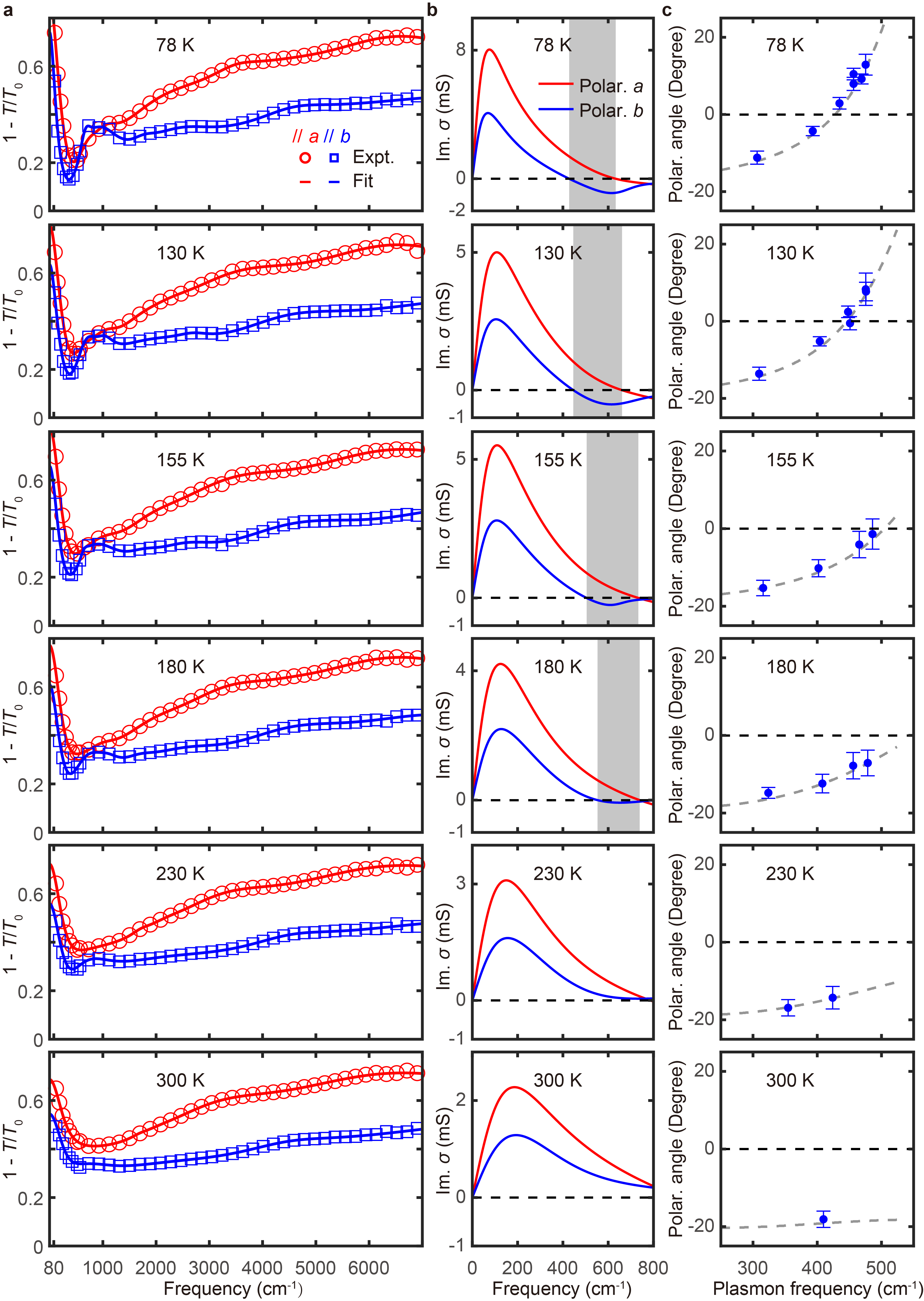


**Fig. S13 | Temperature dependence of OTTs in WTe_2_. a,** The anisotropic extinction spectra and fitting curves of a bare film of WTe_2_ (here with extended frequency range). The sample was maintained at temperatures from 78 K to 300 K from the top to the bottom panel. **b**, The corresponding extracted imaginary parts of the optical conductivities along two crystal axes in **a,** with the hyperbolic regime shaded. **c**, The optimal polarization $\phi_{\max}$ as a function of the plasmon frequency at the corresponding temperature.

**References:**

1 Leavitt, R. P. & Little, J. W. Absorption and emission of radiation by plasmons in two-dimensional electron-gas disks. *Physical Review B* **34**, 2450-2457 (1986).

2 Wang, C. *et al*. Van der Waals thin films of WTe_2_ for natural hyperbolic plasmonic surfaces. *Nature Communications* **11**, 1158 (2020).

3 Gonçalves, P. A. D. & Peres, N. M. *An Introduction to Graphene Plasmonics* (World Scientific, 2016).

4 da Jornada, F. H., Xian, L., Rubio, A. & Louie, S. G. Universal slow plasmons and giant field enhancement in atomically thin quasi-two-dimensional metals. *Nature Communications* **11**, 1013 (2020).

5 Song, C. *et al*. Plasmons in the van der Waals charge-density-wave material 2H-TaSe_2_. *Nature Communications* **12**, 386 (2021).
